# Prognostic utility of Fibrosis-4 Index for risk of subsequent liver and cardiovascular events, and all-cause mortality in individuals with obesity and/or type 2 diabetes: a longitudinal cohort study

**DOI:** 10.1016/j.lanepe.2023.100780

**Published:** 2023-12-19

**Authors:** Quentin M. Anstee, Tina L. Berentzen, Louise M. Nitze, Maximilian Jara, Anders B. Jensen, Mette S. Kjær, Kamal K. Mangla, Jens M. Tarp, Kamlesh Khunti

**Affiliations:** a Translational & Clinical Research Institute, Faculty of Medical Sciences, Newcastle University, Newcastle upon Tyne, UK; b Newcastle NIHR Biomedical Research Centre, Newcastle upon Tyne Hospitals NHS Trust, Newcastle upon Tyne, UK; c Novo Nordisk A/S, Søborg, Denmark; d Diabetes Research Centre, University of Leicester, Leicester General Hospital, Leicester, UK

**Keywords:** Cardiovascular events, FIB-4, Liver events, Hepatic decompensation, Metabolic dysfunction-associated steatotic liver disease

## Abstract

**Background:**

The Fibrosis-4 Index (FIB-4) is used as a non-invasive tool for the presence of advanced liver fibrosis in metabolic dysfunction-associated steatotic liver disease and type 2 diabetes. However, evidence for an association between FIB-4 and risk of mortality and/or liver-related clinical outcomes is limited. The aim of this study was to investigate the association between FIB-4 and subsequent liver events, cardiovascular events, and all-cause mortality in individuals with obesity and/or type 2 diabetes examined in routine general practice.

**Methods:**

This was a longitudinal cohort study in which eligible adults had obesity and/or type 2 diabetes and ≥1 FIB-4 score calculable from UK Clinical Practice Research Datalink GOLD after 1 January 2001. No alcohol-related disorders and/or chronic liver diseases (except non-alcoholic fatty liver disease) and/or no prescriptions of drugs inducing liver disease were permitted. Individuals were followed until time of first event, 10 years, or 1 January 2020. Analyses were conducted using Aalen-Johansen cumulative incidence functions and Cox proportional hazards models.

**Findings:**

Among 44,481 included individuals (mean age 58·8 years; 54% female), there were 979 liver, 6002 cardiovascular, and 8971 mortality events during the 10 years of follow-up. At 10 years, the cumulative incidence of liver events in the high (>2·67), indeterminate (1·30–2·67), and low (<1·30) baseline FIB-4 risk groups were 15%, 3%, and 1%, respectively. Age- and sex-adjusted hazard ratios (HRs) for liver events were elevated in high (16·46; 95% confidence interval [CI] 13·65–19·85) and indeterminate (2·45; 95% CI 2·07–2·90) versus low FIB-4 risk groups. Similar results were found for cardiovascular events and all-cause mortality. Among 20,433 individuals with ≥2 FIB-4 measurements, increase/decrease in FIB-4 12 months after baseline was directly associated with risk of liver events: compared with individuals with low baseline FIB-4 and no change in FIB-4 (reference), the adjusted HR (95% CI) for those with high baseline FIB-4 was 24·27 (16·98–34·68) with a one-unit FIB-4 increase, and 10·90 (7·90–15·05) with a one-unit decrease.

**Interpretation:**

In addition to its value as a diagnostic tool, FIB-4 has clinical utility as a prognostic biomarker. Sequential measurement provides a pragmatic, tractable monitoring biomarker that refines risk assessment for liver events, cardiovascular events, and mortality.

**Funding:**

Novo Nordisk A/S.

**Research in context panel**

**Evidence before this study**

We searched PubMed for articles published between 1 January 2010 and 31 December 2022 that describe the development, validation, and utility of the Fibrosis-4 Index (FIB-4) in individuals with, or at risk of developing, metabolic dysfunction-associated steatotic liver disease (MASLD). The included search terms were (“Fibrosis-4” or “FIB-4”) AND (“non-alcoholic fatty liver disease” OR “non-alcoholic steatohepatitis” OR “type 2 diabetes” OR “obesity”); the search was restricted to English-language publications. We identified numerous international clinical practice guidelines, which consistently recommend the use of FIB-4 as a diagnostic biomarker to determine the presence of advanced liver fibrosis in individuals with MASLD, or at risk of MASLD (eg, individuals with metabolic risk factors such as obesity or type 2 diabetes). Reassessment via repeat testing of FIB-4 is also recommended every 1–3 years in low-risk cases (depending on disease severity or presence of metabolic risk factors). In addition to its diagnostic utility, our search also returned some population-based studies, which have shown that FIB-4 is associated with risk of subsequent liver-related events and mortality. As such, FIB-4 may also provide a prognostic tool to stratify risk of subsequent liver-related events.

**Added value of this study**

This large prospective population-based study examined the associations between FIB-4, 12-month changes in FIB-4 and subsequent risk of liver events, cardiovascular events, and all-cause mortality in individuals with obesity and/or type 2 diabetes seen in routine general practice in the UK. The results of this study confirm the prognostic utility of FIB-4 for risk of subsequent liver-related events, with a high risk of incident liver events in groups with FIB-4 scores indicating high and indeterminate risk of advanced fibrosis. Furthermore, results from the study show that FIB-4 can be used as a prognostic marker of subsequent cardiovascular events and all-cause mortality. Interestingly, analyses adjusted for cardiovascular risk at baseline indicate that associations between FIB-4 and liver events, cardiovascular events, and all-cause mortality were independent of cardiovascular risk at baseline. A 12-month increase/decrease in FIB-4 was associated with higher/lower risk, respectively, of subsequent clinical events across all baseline FIB-4 groups, highlighting the monitoring potential of FIB-4 across a broad population to identify patients at risk of severe events.

**Implications of all the available evidence**

In a broad at-risk population, FIB-4 has clinical utility in general practice as a prognostic biomarker for risk of subsequent liver events, cardiovascular events, and all-cause mortality. Change in FIB-4 across sequential measurements should comprise part of ongoing patient care to monitor the evolving risk of severe events.

## Introduction

Metabolic dysfunction-associated steatotic liver disease (MASLD), formally known as non-alcoholic fatty liver disease (NAFLD) and non-alcoholic steatohepatitis^1^ is a chronic liver disease, the prevalence of which has increased dramatically in recent years.^2^ Metabolic dysfunction-associated steatohepatitis (MASH), the more aggressive form of MASLD, is characterised by inflammation of the liver.^3^ Individuals with MASH, especially those with advanced fibrosis, are at risk of life-threatening liver-related complications and cardiovascular disease, and have a high liver-specific and all-cause mortality.^3^, ^4^, ^5^ For example, increasing fibrosis stage is associated with a 5- to 12-fold increase in the relative risk of liver-related events and all-cause mortality.^5^ MASLD is also associated with a variety of cardiometabolic comorbidities, such as type 2 diabetes and obesity, increasing the risk of progression to the above mentioned severe clinical events.^3^^,^^6^, ^7^, ^8^ Biopsy-confirmed liver fibrosis is an important predictor of morbidity and mortality in individuals with MASLD, but biopsies are invasive, not pragmatic or scalable for use outside of specialist practice, and subject to sampling error and inter-observer variability.^7^^,^^9^, ^10^, ^11^ Fibrosis-4 Index (FIB-4) is a simple non-invasive tool developed to determine the presence of advanced liver fibrosis, with scores categorised into low (<1·30), indeterminate (1·30–2·67), or high (>2·67) risk of fibrosis.^12^^,^^13^ The FIB-4 has recently been shown to perform similarly or better than a range of fibrosis biomarkers including the enhanced liver fibrosis (ELF™) test.^12^^,^^13^ It is consistently recommended by international guidelines as part of first-line assessments in MASLD and type 2 diabetes.^3^^,^^14^, ^15^, ^16^, ^17^ Guidelines also recommend repeat FIB-4 testing every 1–3 years (depending on disease severity or presence/absence of cardiometabolic risk factors) to reassess risk of clinical events.^3^^,^^14^^,^^16^^,^^17^ Some studies have shown an association between FIB-4 and risk of mortality and/or liver-related clinical outcomes in MASLD,^9^^,^^18^, ^19^, ^20^, ^21^ but studies have been limited by small sample sizes, highly selected populations from specialist hepatology clinics, and the omission of cardiovascular outcomes. Studies in a routine primary care setting, which would be optimal for supporting clinical guideline recommendations in a real-world setting, are scarce.

The primary aim of this study was to investigate the association between FIB-4 and subsequent liver events, cardiovascular events, and all-cause mortality in individuals with obesity and/or type 2 diabetes who had measurements available for the calculation of FIB-4 while seen in routine general practice in the UK. The secondary aim was to examine whether assessment of change in FIB-4 score was associated with these clinical events.

## Methods

This longitudinal, observational cohort study followed a protocol approved by the Independent Scientific Advisory Committee of the Clinical Practice Research Datalink (CPRD; protocol number 21_000474) and is reported according to STROBE/RECORD reporting guidelines for observational studies (see the Supplementary appendix for completed STROBE checklist).^22^^,^^23^

### Study objectives

The primary objective was to investigate the association between FIB-4 and time to first liver event, first cardiovascular event, and all-cause mortality among individuals with obesity and/or type 2 diabetes. The secondary objective was to investigate the association between 12-month changes in FIB-4 and time to first liver event, first cardiovascular event, and all-cause mortality.

Five other non-invasive scores were also investigated, but the focus of this study was FIB-4, in line with current clinical guidance.^3^^,^^14^, ^15^, ^16^, ^17^ See the Supplementary Methodology and Supplementary Results for further details.

### Data sources

Data were extracted from the CPRD GOLD, a large database of electronic medical records derived from UK primary care. CPRD is considered broadly representative of the UK population in terms of age, sex, body mass index (BMI), and ethnicity.^24^ CPRD primary care data were linked with Hospital Episode Statistics (HES) admitted and outpatient care data and Office for National Statistics (ONS) death registration data. All included individuals were permanently registered in the CPRD and were followed for an up-to-standard follow-up period.

### Study population

The study population consisted of all acceptable individuals registered in CPRD who were aged ≥18 years with obesity (BMI ≥30 kg/m^2^) and/or type 2 diabetes and had measurements available for FIB-4 calculation after 1 January 2001. BMI was used as recorded or calculated from weight and height using the latest measurement in the last year prior to baseline (date of first FIB-4 measurement after 1 January 2001 and all eligibility criteria being met). Type 2 diabetes was defined from read codes registered in CPRD prior to or at baseline. Individuals with alcohol-related disorders and/or chronic liver disease other than MASLD registered in HES, or with prescriptions of drugs inducing liver disease registered in CPRD, prior to or at baseline, were excluded (see Supplementary Table S1 for exclusion criteria based on CPRD [drug ingredients] and HES [International Classification of Diseases 10 (ICD-10) codes]). Patients with pre-existing registrations of the outcome events (liver or cardiovascular) were excluded from each respective analysis.

The study population for the secondary objective consisted of all individuals in the primary population that had at least one additional FIB-4 measurement taken at 12 (±3) months after the first FIB-4 measurement. Patients with outcome events prior to the second FIB-4 measurement were excluded.

### FIB-4 and changes in FIB-4

FIB-4 was calculated as:^13^ age (years) × aspartate aminotransferase (AST [U/L])/(platelets [10^9^/L] × alanine aminotransferase [ALT {U/L}]^1/2^), with AST and ALT measured on the same day and platelets within ±30 days. FIB-4 was categorised as low (<1·30), indeterminate (1·30–2·67), or high (>2·67) risk of advanced fibrosis using cut-offs previously shown to be associated with advanced fibrosis and in alignment with clinical guidelines.^14^^,^^16^^,^^21^ Changes in FIB-4 were calculated relative to baseline with the second measurement after 12 (±3) months. Extreme values were removed in the analyses: these included AST and ALT values >30,000 U/L, platelet levels >2000 × 10^9^/L, and FIB-4 changes >10.

### Endpoints

Three composite endpoints were defined: time to first liver event (liver-related hospitalisation or death from: hepatocellular carcinoma, liver transplant, chronic liver failure, liver cirrhosis, portal hypertension, or hepatic decompensation [ascites, transjugular intrahepatic portosystemic shunt procedure, hepatorenal syndrome, hepatic encephalopathy, gastro-oesophageal varices with/without bleeding]); time to first cardiovascular event (cardiovascular-related hospitalisation or death from: stroke, acute myocardial infarction, unstable angina, heart failure, and coronary revascularisation); and time to all-cause mortality (death record by any cause). Events were identified via linkage to the ONS and HES using ICD-10 or Office of Population Censuses and Surveys Classification of Interventions and Procedures version 4 codes (Supplementary Table S2).

### Statistical analyses

Statistical analyses were performed using R version 4.1.1 with the following packages: survival, survminer (Cox, Aalen-Johansen, Kaplan-Meier), emmeans (confidence intervals [CI] for estimates), CVrisk (Framingham score). In all analyses, eligible individuals were followed from baseline, ie, their first calculable FIB-4 measurement after 1 January 2001 (primary objective) or second calculable FIB-4 measurement after 1 January 2001 (secondary objective) until time of first event, 10 years’ follow-up or 1 January 2020, whichever came first.

For FIB-4 at baseline, Aalen-Johansen cumulative incidence functions were calculated and plotted according to FIB-4 risk category (low, indeterminate, or high) for liver events, cardiovascular events, and all-cause mortality. All-cause mortality was included as a competing risk factor for liver and cardiovascular events. Hazard ratios (HRs) and 95% CIs were estimated using Cox proportional hazard models with time since first FIB-4 measurement as the underlying timescale using the low FIB-4 category (<1·30) as reference. Crude and adjusted (for sex and age) HRs were estimated.

For 12-month change in FIB-4, Aalen-Johansen cumulative incidence functions were calculated and plotted according to an increase or decrease (of any magnitude) in FIB-4 in each of the three baseline FIB-4 categories. Crude and adjusted (sex and age) Cox models including change in FIB-4 (continuous) and baseline FIB-4 (categorical) were made. To assess whether associations between changes in FIB-4 and liver events, cardiovascular events, and all-cause mortality were independent of baseline FIB-4, a model was fitted with an interaction term of 12-month change in FIB-4 and baseline FIB-4. Estimated HRs were plotted using individuals with low baseline FIB-4 and no change in FIB-4 as reference (HR = 1). Analyses were repeated using changes in FIB-4 after 6 (±3) and 36 (±6) months.

The proportional hazards assumption was evaluated based on the Schoenfeld residuals and linearity of continuous variables was assessed using restricted cubic splines.

### Analyses adjusted for cardiovascular risk at baseline (FIB-4 at baseline)

To evaluate if FIB-4 was associated with liver events, cardiovascular events, and all-cause mortality independent of cardiometabolic risk, supplementary analyses were conducted in a subpopulation of individuals for whom the Framingham risk score could be calculated based on the methodology published by D’Agostino et al.^25^ All Cox models were repeated in this subpopulation with an additional key analysis adjusting for the Framingham risk score measured at baseline. To further investigate the cardiometabolic risk, all these analyses were repeated with the SCORE2/SCORE2-OP^26^^,^^27^ instead of the Framingham score.

### Supplementary analyses (FIB-4 at baseline)

To evaluate if associations depended on age, the age-dependent FIB-4 cut-offs suggested by McPherson et al.^28^ were investigated. In these analyses, individuals aged ≤35 years were excluded and cut-offs for individuals aged ≥65 years were changed (low: <2·0; indeterminate: 2·0–2·67; high: >2·67). Cut-offs for individuals aged 36–64 years were unchanged.

To evaluate if undiagnosed diseases affected associations (reverse causality) and/or associations were attenuated over time, analyses excluding individuals with events in the first 6 months, or the first 12 months of follow-up, and analyses with follow-up ending at 2·5 and 5 years, were performed.

### Subpopulation analyses (FIB-4 at baseline)

To assess whether associations differed for individuals with obesity and for those with type 2 diabetes, analyses were repeated in populations with obesity, with type 2 diabetes, and with both obesity and type 2 diabetes.

### Role of the funding source

The sponsor, with input from authors, was responsible for the study design; preparation of the study protocol; analysis of the data and interpretation of the results. The sponsor also contributed to the writing of the report and to the decision to submit the paper for publication.

## Results

### Study flow, baseline demographics, and clinical characteristics

**Fig. 1:**
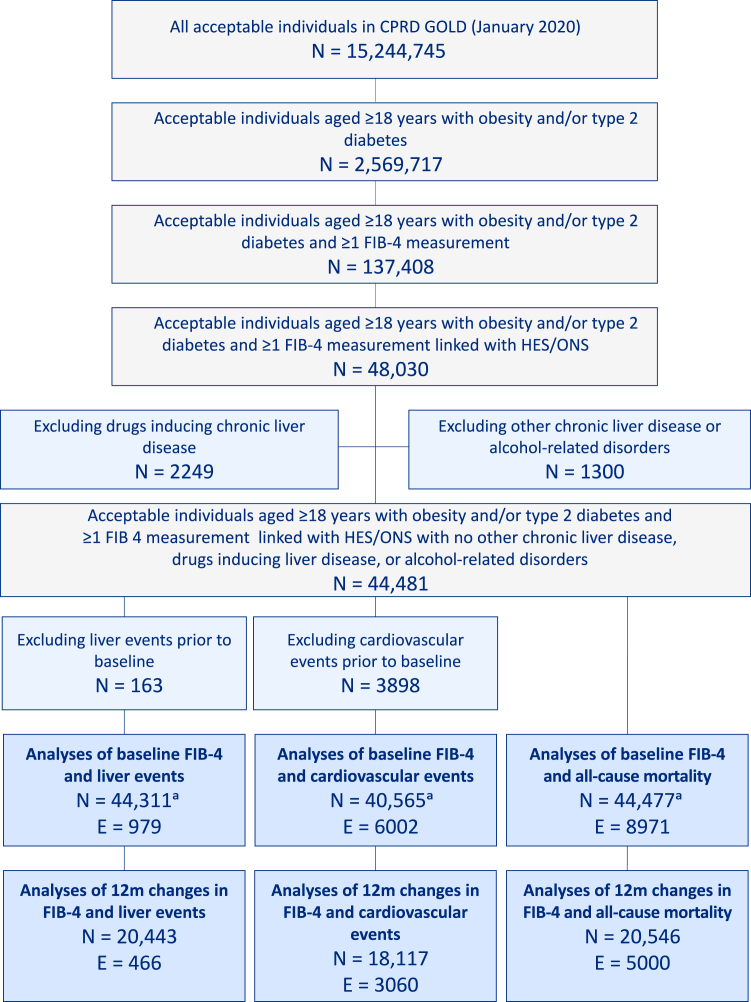
Flowchart. CPRD = Clinical Practice Research Datalink. E = events. FIB-4 = Fibrosis-4 Index. HES = Hospital Episode Statistics. m = month. ONS = Office of National Statistics. ^a^Individuals with no follow-up time (liver: n = 7; cardiovascular: n = 18; mortality: n = 4), defined as individuals with an event occurring on the date of FIB-4 measurement (ie, baseline) were excluded from analyses.

A total of 2,569,717 acceptable individuals aged ≥18 years with obesity and/or type 2 diabetes were present in CPRD GOLD; of these, 137,408 had available measures for FIB-4 calculation (Fig. 1). Excluding those without linkage to HES/ONS, those who received drugs inducing chronic liver disease, those with chronic liver disease other than MASLD, and/or those with alcohol-related disorders, left a study population of 44,481. After excluding individuals with prior events and individuals with no follow-up time, analyses of liver events, cardiovascular events, and all-cause mortality were conducted in 44,311, 40,565, and 44,477 individuals, respectively (Fig. 1). Individuals with one baseline FIB-4 measure and a second FIB-4 measure after 12 (±3) months were eligible for analyses of 12-month changes in FIB-4. After excluding individuals with events prior to (or on the day of) their second FIB-4 measure, analyses of liver events, cardiovascular events, and all-cause mortality were conducted in 20,443, 18,117, and 20,546 individuals, respectively (Fig. 1).

Baseline characteristics are shown in Table 1. Of the eligible individuals, 54% were female and 44% had type 2 diabetes, with a median age of 58·8 years and BMI of 32·1 kg/m^2^. The median FIB-4 values at baseline were 0·8, 1·7, and 3·4 in the low, indeterminate, and high FIB-4 groups, respectively. As expected, individuals in the high FIB-4 group were older, more often male, had a higher prevalence of type 2 diabetes, and had higher serum ALT and AST than the indeterminate and low FIB-4 categories. Baseline characteristics in the 12-month change in FIB-4 population are shown in Supplementary Table S3. Individuals with repeated measures were slightly older, had slightly higher medication use and more hospitalisations for comorbidities than those in the primary population. Baseline characteristics overall and by baseline FIB-4 in individuals without linked HES/ONS data (ie, with general practitioner [GP] observed data only) were not notably different to the linked CPRD/HES populations (Supplementary Table S4).

**Table 1:** Baseline demographics and clinical characteristics.

Baseline parameter	FIB-4 low (<1·30)	FIB-4 indeterminate (1·30–2·67)	FIB-4 high (>2·67)	Overall (N = 44,481)	*P* value^b^ (Indeterminate FIB-4 versus low FIB-4/high FIB-4 versus low FIB-4)
N	29,359	13,189	1933	44,481	
**Patient characteristics**					
Female, %	58	46	40	54	<0·01
Age, years	52·0 (26·6, 76·2)	70·6 (50·4, 87·2)	74·3 (50·5, 90·6)	58·8 (29·5, 83·7)	<0·01
Race, %					
White	83	88	88	85	<0·01
Asian	4	1	2	3	<0·01
Black	2	2	3	2	1·0/<0·01
Unknown or missing	7	5	4	7	<0·01
BMI, kg/m^2^	32·7 (25·1, 44·4)	31·2 (22·6, 40·8)	30·8 (21·6, 40·9)	32·1 (23·8, 43·3)	<0·01
Type 2 diabetes, %	37	56	64	44	<0·01
Duration, years	0 (0·0, 9·8)	0 (0·0, 15·2)	0·3 (0·0, 16·1)	0 (0·0, 12·1)	
Framingham risk score^a^	18·3 (4·9, 51·7)	24·7 (9·3, 58·7)	23·9 (7·5, 58·1)	20·2 (5·5, 54·3)	<0·01
Smoking status^a^^,^^c^					
Current smoker	19	10	10	16	<0·01
Ex-smoker	22	32	32	26	<0·01
Never smoker	35	35	35	35	0·97/0·79
**Liver parameters**					
AST, U/L	22 (14, 42)	25 (16, 60)	38 (19, 212)	24 (15, 53)	<0·01
ALT, U/L	26 (12, 71)	24 (11, 80)	30 (10, 202)	26 (12, 78)	<0·01
Platelets, 10^9^/L	280 (197, 415)	218 (153, 311)	157 (70, 267)	257 (160, 394)	<0·01
**Metabolic parameters**					
HbA_1c_,^a^%	7·1 (5·5, 11·2)	6·8 (5·5, 10·0)	6·7 (5·3, 9·8)	6·9 (5·5, 10·8)	<0·01
Creatinine, μmol/L	80 (56, 115)	89 (61, 145)	90 (59, 172)	83 (57, 127)	<0·01
HDL,^a^ mmol/L	1·2 (0·8, 1·9)	1·3 (0·8, 2·0)	1·2 (0·7, 2·1)	1·2 (0·8, 2·0)	<0·05
LDL,^a^ mmol/L	3·0 (1·5, 4·9)	2·6 (1·3, 4·5)	2·4 (1·1, 4·3)	2·9 (1·4, 4·8)	<0·01
Triglycerides,^a^ mmol/L	1·6 (0·7, 4·1)	1·5 (0·7, 3·6)	1·4 (0·7, 3·4)	1·6 (0·7, 3·9)	<0·01
**Hospitalisation for comorbidities, %**					
Hypertension	45	68	68	53	<0·01
Dyslipidaemia	23	35	31	27	<0·01
Chronic kidney disease	9	20	21	13	<0·01
**Prescribed medication, %**					
Anti-hypertensive	24	45	50	31	<0·01
Metformin	18	23	23	19	<0·01
Lipid-lowering medication	27	49	47	34	<0·01

Values are median (5th percentile, 95th percentile) unless otherwise stated.

ALT = alanine aminotransferase. AST = aspartate aminotransferase. BMI = body mass index. FIB-4 = Fibrosis-4 Index. HbA_1c_ = glycated haemoglobin. HDL = high-density lipoprotein. LDL = low-density lipoprotein.

a Missing for ≥20% of the population.

b For continuous variables, Welsch two sample t-test for difference in means of indeterminate and high FIB-4 versus low FIB-4; for categorical variables, Pearson's chi-squared test for low versus indeterminate FIB-4 and low versus high FIB-4.

c As reported by the general practitioner with no time threshold.

### Clinical events by baseline FIB-4

The 44,311, 40,565, and 44,477 individuals in the analyses of liver events, cardiovascular events, and all-cause mortality were each followed for a median time of 10·0 person-years. During follow-up, 979 liver events, 6002 cardiovascular events, and 8971 deaths were registered. The most common liver events were ascites, cirrhosis, or gastro-oesophageal varices, and the most frequent cardiovascular events were heart failure and stroke.

**Fig. 2:**
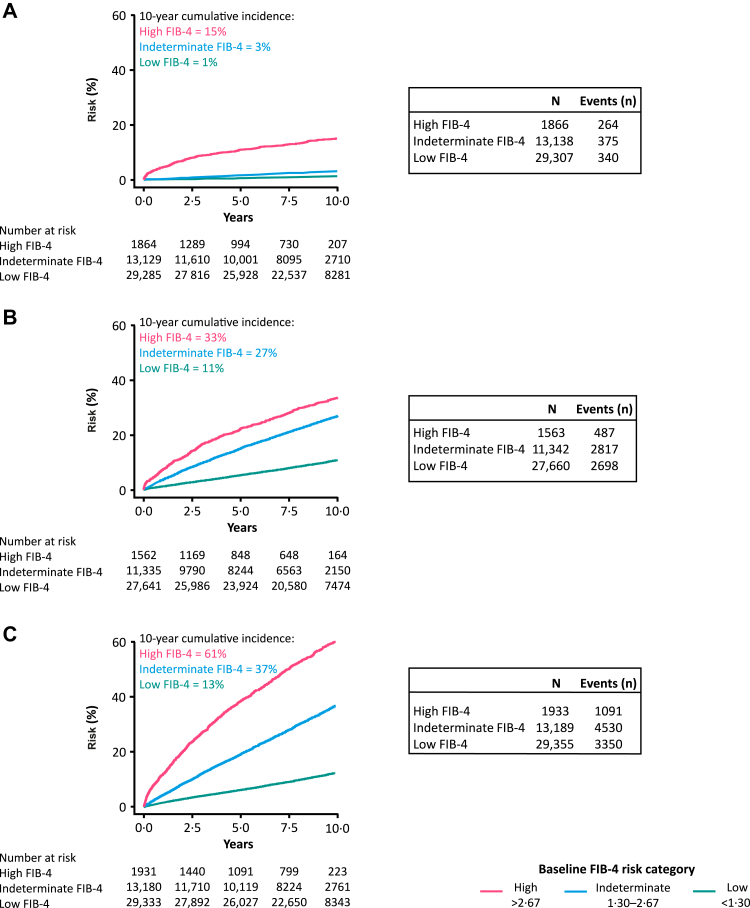
Cumulative incidence according to baseline FIB-4 for A) liver events, B) cardiovascular events, C) all-cause mortality. Event risks plotted as Aalen-Johansen cumulative incidence functions, with all-cause mortality included as a competing risk factor in plots of liver and cardiovascular events. FIB-4 risk categories: low <1·30; indeterminate 1·30–2·67; high >2·67. FIB-4 = Fibrosis-4 Index.

After 10 years of follow-up, the cumulative incidence of liver events was 15%, 3%, and 1% in the high, indeterminate, and low baseline FIB-4 risk strata, respectively (Fig. 2). The cumulative incidence of cardiovascular events and all-cause mortality was also greatest in individuals with high baseline FIB-4 and lowest in individuals with low FIB-4 (Fig. 2).

HRs according to baseline FIB-4 are presented in Table 2. Baseline FIB-4 was associated with liver events, with substantially elevated HRs for both high (16·46; 95% CI 13·65–19·85) and indeterminate (2·45; 95% CI 2·07–2·90) versus low FIB-4 groups in the age- and sex-adjusted model. Associations were also seen between baseline FIB-4 and cardiovascular events and all-cause mortality. The age- and sex-adjusted HRs for the high versus low FIB-4 group were 1·34 (95% CI 1·21–1·48) for cardiovascular events and 1·56 (95% CI 1·45–1·68) for all-cause mortality.

**Table 2:** Hazard ratios of liver events, cardiovascular events, and all-cause mortality by baseline FIB-4.

FIB-4 category	Patients (n)	Events (n)	Median follow-up (days)	Crude HR (95% CI)	Adjusted (age and sex) HR (95% CI)
**Liver events**					
Overall	44,311	979	3652		
FIB-4 low	29,307^a^	340	3652	1·00	1·00
FIB-4 indeterminate	13,138	375	3316	2·81 (2·43–3·26)	2·45 (2·07–2·90)
FIB-4 high	1866	264	2106	18·42 (15·67–21·65)	16·46 (13·65–19·85)
**Cardiovascular events**					
Overall	40,565	6002	3652		
FIB-4 low	27,660^a^	2698	3652	1·00	1·00
FIB-4 indeterminate	11,342	2817	3121	2·97 (2·82–3·13)	1·01 (0·95–1·07)
FIB-4 high	1563	487	2146	4·73 (4·29–5·21)	1·34 (1·21–1·48)
**All-cause mortality**					
Overall	44,477	8791	3652		
FIB-4 low	29,355^a^	3350	3652	1·00	1·00
FIB-4 indeterminate	13,189	4530	3350	3·44 (3·29–3·59)	0·97 (0·93–1·02)
FIB-4 high	1933	1091	2271	7·25 (6·77–7·77)	1·56 (1·45–1·68)

HRs and 95% CI were estimated using Cox proportional hazard models with time since first FIB-4 measurement as the underlying timescale. Crude results and results adjusted for sex and age at baseline are presented. FIB-4 score at baseline was categorised based on risk of advanced fibrosis as low (<1·30), indeterminate (1·30–2·67), or high (>2·67) risk.

CI = confidence interval. FIB-4 = Fibrosis-4 Index. HR = hazard ratio.

a One individual in the FIB-4 low group had no information on sex and was therefore excluded from age- and sex-adjusted analyses.

Results from supplementary analyses of FIB-4 using age-adjusted cut-off for individuals aged ≥65 years showed that the age- and sex-adjusted HRs for the high versus low FIB-4 group were 12·40 (95% CI 10·51–14·64) for liver events, 1·33 (95% CI 1·21–1·47) for cardiovascular events, and 1·58 (95% CI 1·48–1·69) for all-cause mortality. Likewise, results from supplementary analyses of FIB-4 investigating reverse causality or whether associations were attenuated over time, were not notably different to those from the primary analyses (see Supplementary Results for further details).

### Analyses adjusted for cardiovascular risk at baseline

Results of baseline FIB-4 in the FIB-4 subpopulation with an available Framingham cardiovascular risk score at baseline were not notably different to results seen in the primary population, and there was no notable impact on the associations after further adjustment for the Framingham risk score (Table 3) or when using the SCORE2 (data not shown).

**Table 3:** Hazard ratios of liver events, cardiovascular events, and all-cause mortality by baseline FIB-4 in the subpopulation with Framingham risk score available.

FIB-4 category	Patients (n)	Events (n)	Median follow-up (days)	Crude HR (95% CI)	Adjusted (age, sex) HR (95% CI)	Adjusted (age, sex, FramH) HR (95% CI)
**Liver events**						
Overall	17,680	404	3652			
FIB-4 low	12,452	145	3652	1·00	1·00	1·00
FIB-4 indeterminate	4717	150	3652	2·86 (2·28–3·59)	2·76 (2·15–3·55)	2·76 (2·15–3·55)
FIB-4 high	511	109	2858	23·80 (18·56–30·53)	23·08 (17·57–30·32)	23·09 (17·58–30·34)
**Cardiovascular events**						
Overall	16,226	1974	3652			
FIB-4 low	11,612	1138	3652	1·00	1·00	1·00
FIB-4 indeterminate	4145	750	3612	1·97 (1·80–2·16)	1·11 (1·01–1·23)	1·13 (1·02–1·25)
FIB-4 high	469	86	2886	2·33 (1·87–2·91)	1·26 (1·00–1·57)	1·28 (1·03–1·61)
**All-cause mortality**						
Overall	17,751	2239	3652			
FIB-4 low	12,475	1137	3652	1·00	1·00	1·00
FIB-4 indeterminate	4737	913	3652	2·21 (2·03–2·41)	1·11 (1·01–1·22)	1·12 (1·02–1·23)
FIB-4 high	539	189	2995	4·86 (4·16–5·66)	2·28 (1·94–2·67)	2·32 (1·98–2·72)

Analyses included all individuals with a Framingham cardiovascular risk score available at baseline. HRs and 95% CI were estimated using Cox proportional hazard models with time since first FIB-4 measurement as the underlying timescale. Crude results, results adjusted for sex and age at baseline, and results adjusted for sex, age, and Framingham cardiovascular risk score are presented. Adjustment for Framingham cardiovascular risk score was at the time of FIB-4 measurement. FIB-4 score at baseline was categorised based on risk of advanced fibrosis as low (<1·30), indeterminate (1·30–2·67), or high (>2·67) risk.

CI = confidence interval. FIB-4 = Fibrosis-4 Index. FramH = Framingham. HR = hazard ratio.

### Clinical events according to a 12-month increase or decrease in FIB-4 by baseline FIB-4

In the analyses of the 12-month changes in FIB-4, there were 466 liver events (most commonly ascites, cirrhosis, or gastro-oesophageal varices), 3060 cardiovascular events (most commonly heart failure, stroke, and cardiovascular death), and 5000 deaths during the 10 years of follow-up.

Among the 20,433 individuals in the analyses of liver events, the median FIB-4 score was 1·12 to 1·15 at baseline and follow-up, respectively. During the 12-months, 10·5% and 8·2% of patients increased and decreased FIB-4 category, respectively. To determine a typical FIB-4 change, the absolute change was calculated where both increases and decreases were counted positively. For this, the absolute median FIB-4 change was 0·16.

**Fig. 3:**
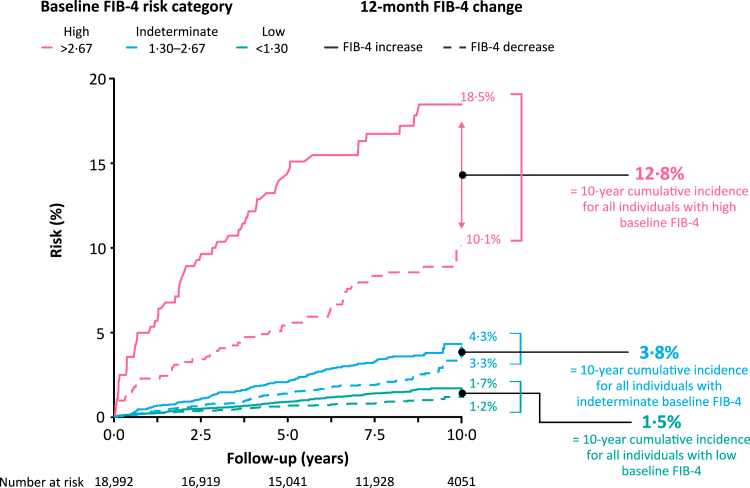
Cumulative incidence of liver events according to a 12-month increase or decrease in FIB-4 by baseline FIB-4 category. Event risks plotted as Aalen-Johansen cumulative incidence functions, with all-cause mortality included as a competing risk factor. FIB-4 risk categories: low < 1·30; indeterminate 1·30–2·67; high >2·67. FIB-4 = Fibrosis-4 Index.

The cumulative incidence of a liver event after 10 years in the high baseline FIB-4 risk group was 12·8% when not stratifying for 12-month increase or decrease in FIB-4. When stratifying, the cumulative incidence was 18·5% and 10·1% for individuals whose FIB-4 increased or decreased, respectively (Fig. 3). A similar pattern was observed for liver events in the indeterminate and low baseline FIB-4 risk groups. The incidence of cardiovascular events and deaths was also consistently higher in those with an increase versus decrease in FIB-4 in the 12 months from baseline (Supplementary Fig. S1).

**Fig. 4:**
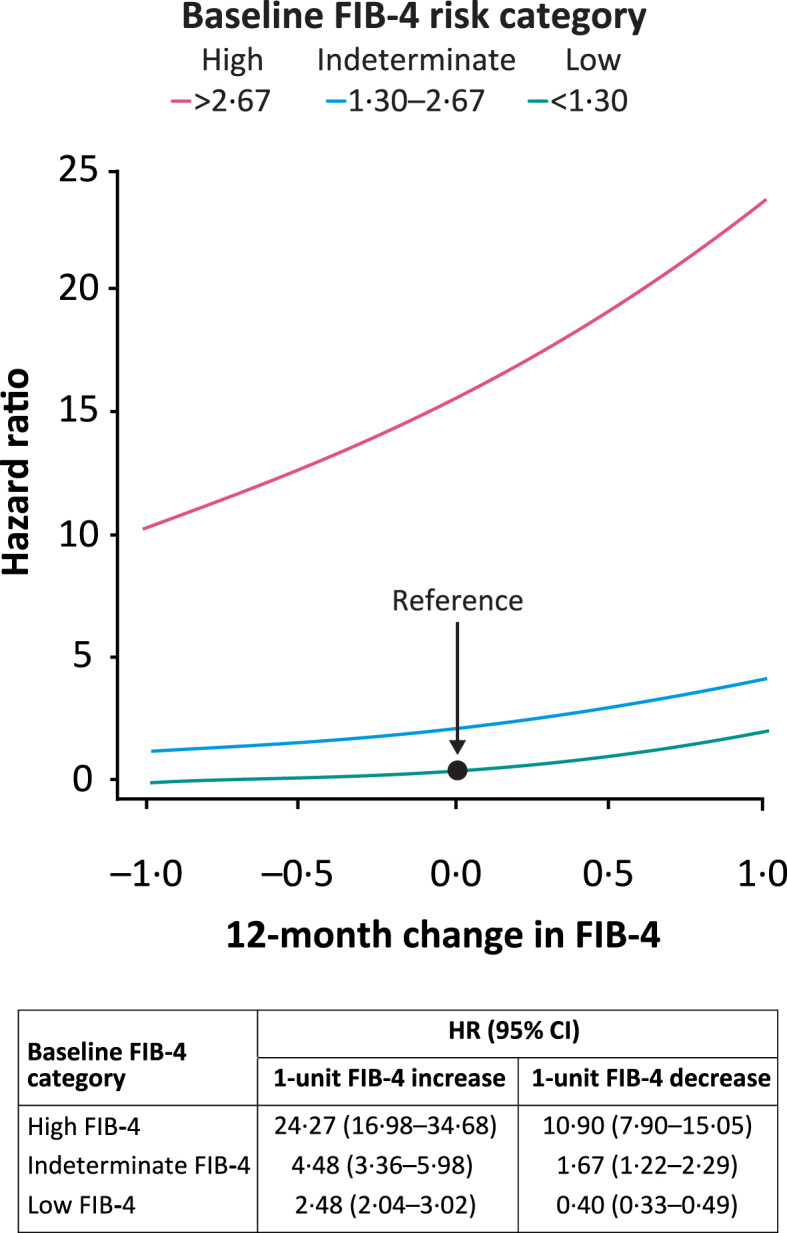
Hazard ratios of liver events for 12-month changes in FIB-4. Reference is patients with low baseline FIB-4 and no change in FIB-4 (HR = 1). Time since FIB-4 measurement as the underlying timescale and age included in strata (baseline hazard). The model included: change in FIB-4 (continuous), baseline FIB-4 (categorical), sex (categorical), and the interaction change in FIB-4 (continuous)∗baseline FIB-4 (categorical). FIB-4 risk categories: low <1·30; indeterminate 1·30–2·67; high >2·67. CI = confidence interval. FIB-4 = Fibrosis-4 Index. HR = hazard ratio.

In Cox models adjusted for age, sex, and baseline FIB-4, change in FIB-4 was directly associated with risk of a liver event (Fig. 4). The interaction model showed that slopes for the impact of a FIB-4 unit change on the HRs of liver events varied across baseline FIB-4 risk groups, implying that the association was dependent on baseline FIB-4. Thus, compared with individuals with low baseline FIB-4 and no change in FIB-4 (reference), the HR was 24·27 (95% CI 16·98–34·68) for those with high baseline FIB-4 and a one-unit FIB-4 increase, and 10·90 (7·90–15·05) for those with high baseline FIB-4 with a one-unit decrease (Fig. 4). Compared with the reference, those with indeterminate and low baseline FIB-4 and one-unit FIB-4 increase/decrease also had significantly higher/lower risk (Fig. 4). A one-unit change in FIB-4 is a large change, and the HRs for median increase (0·16) in FIB-4 from the same models were 1·15 (1·12–1·19), 2·95 (2·32–3·77), and 17·3 (12·9–23·23) for low, indeterminate, and high baseline FIB-4 groups compared with the reference, respectively. HRs for liver events and a 0·5-unit increase in FIB-4 are reported in Supplementary Results.

Similar results were found for the association between change in FIB-4 and cardiovascular events and all-cause mortality in crude analyses, but these associations, particularly for cardiovascular events, were attenuated by adjustment for sex and age (data not shown).

Results from supplementary analyses assessing 6-month change or 36-month change in FIB-4 were overall similar to those from the primary analyses (see Supplementary Results for further details).

## Discussion

The results of this longitudinal, population-based observational cohort study of individuals with obesity and/or type 2 diabetes examined in general clinical practice provides valuable real-world insights about FIB-4. Complementing the existing literature that demonstrates the diagnostic utility of FIB-4,^12^ this study demonstrates its potential across two additional biomarker contexts of use: prognostic and monitoring. The key findings of the study were: (1) FIB-4 was strongly associated with risk of subsequent liver and cardiovascular events, and all-cause mortality; (2) monitoring changes in FIB-4 further refined the prognostic potential, as 12-month increase/decrease in FIB-4 was associated with higher/lower risk of subsequent liver and cardiovascular events, and all-cause mortality across all FIB-4 groups at baseline (see visual abstract/summary figure).

FIB-4 was most prominently associated with liver events, with a very high risk in both the high and indeterminate FIB-4 groups. Associations were also seen for cardiovascular events and all-cause mortality. Adjustment for age and sex had no notable influence on the association with liver events but attenuated associations for cardiovascular events or all-cause mortality, indicating as expected that age explained some of the increased risk of cardiovascular events and all-cause mortality associated with FIB-4. However, further studies are needed to fully understand the impact of age. The current findings are in line with a recent study of patients with MASLD, MASH, or at risk of MASH, which reported that high FIB-4 was associated with major adverse cardiovascular events (HR 1·82; 95% CI 1·63–2·04); however, the study did not report on liver events and the follow-up period was much shorter than the current study at only 3 years.^29^

Our findings support current clinical practice guidelines that uniformly recommend automatic calculation of FIB-4 in individuals with, or at risk of, MASLD (eg, with metabolic risk factors) to rule out advanced fibrosis, allowing low-risk individuals (FIB-4 <1·3) to continue management in primary care.^3^^,^^14^^,^^16^ Guidance from the American Diabetes Association similarly recommends evaluating for the presence of MASH and liver fibrosis in individuals with type 2 diabetes or prediabetes with cardiometabolic risk factors if they have either elevated liver enzymes or fatty liver on imaging, with FIB-4 noted as the initial test of choice.^17^ Guidelines from the European Association for the Study of the Liver (EASL) also recommend use of serum scores such as FIB-4 and vibration-controlled transient elastography (VCTE)-measured liver stiffness (FibroScan™) to stratify risk of liver-related outcomes.^14^ Our results support this recommendation, and demonstrate that it is also relevant to assess FIB-4 in routine clinical care to understand the subsequent risk of cardiovascular events and all-cause mortality. Of note, a considerable proportion of individuals included in the study were in the indeterminate FIB-4 category, and this diagnostic ‘grey zone’ is a recognised limitation of the FIB-4. Per clinical guideline recommendations, patients with indeterminate FIB-4 scores in primary care are candidates for further tests including liver stiffness measurement or ELF™ test, followed by referral to secondary care when clinically indicated.^3^^,^^14^ The need for further evaluation of such cases is supported by our results, which suggest that individuals in the indeterminate FIB-4 group have an increased risk of liver-related events.

Current clinical practice guidelines recommend reassessment by repeating FIB-4 every 1–3 years in patients initially stratified as low risk, but there is limited evidence on the optimal interval and the clinical rationale for the retesting period varies across different guidelines.^3^^,^^14^^,^^17^ For example, EASL recommendations for retesting are based on disease severity, while American Association for the Study of Liver Diseases recommendations are based on presence of metabolic risk factors, and American Gastroenterological Association guidance depends on a clinical change in circumstance such as incident type 2 diabetes.^3^^,^^14^^,^^17^ Our study demonstrates that a 12-month increase/decrease in FIB-4 is associated with higher/lower risk of liver events across the FIB-4 baseline groups. These results, which are in accordance with findings from Hagstrøm et al.,^30^ highlight the monitoring potential of FIB-4 and indicate that retesting may be beneficial. Associations when the time interval for FIB-4 retesting was 6 months or 36 months were similar to 12 months (data not shown). The absolute change in FIB-4 in our data was, however, modest and only half of our population had repeated measures (albeit over 20,000 individuals), so further independent validation would be beneficial to substantiate these findings.

Cardiovascular disease is an important cause of morbidity and mortality in individuals with MASLD.^3^^,^^31^^,^^32^ We therefore explored the impact of the cardiovascular risk at baseline (as measured by Framingham risk score^25^) on the studied associations and found that FIB-4 was associated with liver events, cardiovascular events, and all-cause mortality after adjustment for cardiovascular risk at baseline. These analyses were conducted in a selected subpopulation with the Framingham risk score available, but still the limited impact of the cardiovascular adjustment on associations highlights the prognostic potential of FIB-4.

This study has several strengths and limitations. Major strengths were the prospective design, large sample size, a representative population-based primary care sample, long duration of follow-up, and adequate statistical power to investigate the long-term risk of clinical events. Patients were followed from their first measurement of FIB-4 and onwards through the efficient UK hospital and death registries. The linkage of GP data with these registries implied some attrition of our cohort, which mostly relates to administrative issues,^24^ minimising the risk of selection bias. This is supported by the fairly similar baseline characteristics across the GP-only and linked GP/HES populations. The liver function tests used for the FIB-4 calculation were made by GPs prior to, and independently of, a later diagnosis of a clinical event, and clinical events were ascertained using routinely collected data in the UK registries. Although misclassification can occur in registries, most of our events were likely captured correctly due to their nature and severity, which altogether minimises the risk of information bias. However, since bias from unrecognised disease can never be excluded, we conducted analyses where events in the first 6 (and 12) months of follow-up were excluded and where follow-up was restricted to the first 2·5 (and 5) years. These exclusions/restrictions had no notable influence on the associations. Although the undertaking of liver function tests by GPs represents a strength of the present study, it is also limited by a lack of recorded information on the clinical justification for taking liver function measurements. Only a small proportion (5·7%) of the individuals with obesity and/or type 2 diabetes in CPRD had the relevant liver function tests for FIB-4 recorded. We assume that GPs have performed these tests on individuals they considered to be at risk of liver disease, which is also reflected in the high cumulative incidences of liver events in the study. These findings strongly highlight the need for increased measurement and use of FIB-4 in routine clinical practice. Additionally, this study evaluated only simple serum scores that are calculable from electronic medical records. Real-world investigations of more advanced guideline-recommended tests, such as VCTE, are challenging at present due to limited usage in clinical practice^33^ but should be considered for future studies. Finally, the possibility of residual confounding and/or confounding from other factors cannot be fully excluded.

Recently, a new nomenclature for NAFLD has been proposed by EASL-AASLD-APASL in a multi-society Delphi consensus statement.^1^ Building on the previous proposal to update NAFLD to metabolic-associated fatty liver disease with a focus on positive metabolic criteria versus an exclusionary diagnosis,^34^ MASLD encompasses patients who have hepatic steatosis diagnosed histologically or by imaging and ≥1 of five cardiometabolic risk factors.^1^ In the current study, we were unable to fully apply the new MASLD criteria as steatosis was not assessed. However, patients did have ≥1 cardiometabolic criteria (type 2 diabetes or obesity) for MASLD, and other causes of steatosis such as alcohol-related disorders, chronic liver disease, and drugs inducing liver disease were excluded. Moreover, as approximately 70% of patients with overweight or obesity have MASLD,^35^ the current patient sample would be considered at risk of MASLD and is likely to be heavily enriched for patients with MASLD, reinforcing the clinical relevance of these findings going forward, accepting that presence of hepatic steatosis could not be directly assessed.

### Conclusions

It is now recognised that there is significant overlap in MASLD (formally NAFLD) and type 2 diabetes, with shared risk factors and underlying pathophysiological mechanisms. The current results align with MASLD and type 2 diabetes clinical practice guidelines that recommend the use of FIB-4 as a first-line clinical decision aid in routine primary care to detect advanced fibrosis and aid risk stratification.^3^^,^^14^^,^^16^^,^^17^ In addition, our results indicate that an increase in FIB-4 over 12 months is associated with a higher risk of clinical events, suggesting that sequential testing of FIB-4 should be incorporated into clinical management for monitoring the long-term risk of adverse outcomes. The use of FIB-4 is a valuable tool for counselling patients, through assessing fibrosis severity, monitoring progression, and predicting the risk of long-term outcomes.

## Contributors

QMA and KK were involved in the study concept and design. TLB, LMN, MJ, ABJ, MSK, KKM, and JMT were involved in the study concept and design, and analysis of data, and all authors were involved in interpretation of the study data. TLB, JMT, and ABJ accessed and verified the underlying study data. JMT built the patient cohort. JMT and ABJ carried out the statistical analysis. The manuscript was written and edited by medical writers, under the direction of the authors. All authors contributed to the drafting and critical revision of the manuscript, provided final approval, and were responsible for the decision to submit the manuscript.

## Data sharing statement

Data will be shared with bona fide researchers who submit a research proposal approved by an independent review board. Individual patient data will be shared in datasets in a de-identified and anonymised format. Data will be made available after research completion. Information about data access request proposals can be found at novonordisk-trials.com.

## Declaration of interests

QMA is coordinator of the IMI2 Liver Investigation: Testing Marker Utility in Steatohepatitis (LITMUS) consortium, which is funded by the European Union Horizon 2020 programme and the European Federation of Pharmaceutical Industries and Associations (EFPIA). This multi-stakeholder consortium includes industry partners. He reports research grant funding from AstraZeneca, Boehringer Ingelheim, and Intercept; consultancy on behalf of Newcastle University for Alimentiv, Akero, AstraZeneca, Axcella, 89Bio, Boehringer Ingelheim, Bristol Myers Squibb, Galmed, Genfit, Genentech, Gilead, GSK, Hanmi, HistoIndex, Intercept, Inventiva, Ionis, IQVIA, Janssen, Madrigal, Medpace, Merck, NGMBio, Novartis, Novo Nordisk, PathAI, Pfizer, Prosciento, Poxel, Resolution Therapeutics, Roche, Ridgeline Therapeutics, RTI, Shionogi, and Terns; speaker fees/honoraria from Fishawack, Integritas Communications, Kenes, Novo Nordisk, Madrigal, Medscape, and Springer Healthcare; and royalties from Elsevier.

MJ, MSK, TLB, LMN, JMT, KKM, and ABJ are full-time employees and shareholders of Novo Nordisk A/S.

KK has acted as consultant, advisory board member, and speaker for Abbott, Amgen, AstraZeneca, Bayer, Boehringer Ingelheim, Eli Lilly, Merck Sharp & Dohme, Novo Nordisk, Roche, Sanofi-Aventis, and Servier; and received EACME grants from AstraZeneca, Boehringer Ingelheim, Eli Lilly, Merck Sharp & Dohme, Novartis, Novo Nordisk, and Sanofi-Aventis.

## Appendix A Supplementary data

Supplementary Material

Cover letter

Graphical abstract
