# Supplementary material for: Prognostic utility of Fibrosis-4 Index for risk of subsequent liver and cardiovascular events, and all-cause mortality in individuals with obesity and/or type 2 diabetes: a longitudinal cohort study
Source: Lancet Reg Health Eur. 2023 Dec 19;36:100780. doi: 10.1016/j.lanepe.2023.100780 (PMC10769893; doi:10.1016/j.lanepe.2023.100780)
Supplement: Supplementary Material [file mmc1.docx]

# Supplementary appendix

## Table of contents

[Supplementary appendix 1](#_Toc146111707)

[Table of contents 1](#_Toc146111708)

[Supplementary methodology 2](#_Toc146111709)

[Supplementary results 3](#_Toc146111710)

[Supplementary analyses (FIB-4 at baseline) 3](#_Toc146111711)

[Supplementary analyses (change in FIB-4) 3](#_Toc146111712)

[*Supplementary Table 1:* Codes used to identify criteria for exclusion in CPRD, HES ADMIN, and HES OP 4](#_Toc146111713)

[*Supplementary Table 2:* Codes used to identify liver events, CV events, and all-cause mortality in ONS, HES ADMIN, and HES OP 5](#_Toc146111714)

[*Supplementary Table 3:* Baseline demographics and clinical characteristics in the baseline and 12-month change analyses 6](#_Toc146111715)

[*Supplementary Table 4:* Baseline demographics and clinical characteristics (GP observed data*) 7](#_Toc146111716)

[*Supplementary Table 5:* Hazard ratios of liver events, CV events, and all-cause mortality by baseline FIB-4 using age-dependent FIB-4 cut-offs 8](#_Toc146111717)

[*Supplementary Table 6:* Hazard ratios of liver events, CV events, and all-cause mortality by baseline FIB-4, excluding patients with events occurring in the first 6 months or first 12 months 9](#_Toc146111718)

[*Supplementary Table 7:* Hazard ratios of liver events, CV events, and all-cause mortality by baseline FIB-4 with 2·5 years or 5 years follow-up 10](#_Toc146111719)

[*Supplementary Table 8:* Hazard ratios of liver events, CV events, and all-cause mortality by baseline non-invasive score 11](#_Toc146111720)

[*Supplementary Table 9:* Hazard ratios of liver events, CV events, and all-cause mortality by baseline non-invasive scores in the subpopulations with a Framingham risk score available 13](#_Toc146111721)

[*Supplementary Table 10:* Hazard ratios of liver events for 12-month, 0·5 unit increase in FIB-4. 15](#_Toc146111722)

[*Supplementary Figure 1:* Cumulative incidence of (A) CV events and (B) all-cause mortality according to a 12-month increase or decrease in FIB-4 by baseline FIB-4 category 16](#_Toc146111723)

[Supplementary reference 23](#_Toc146111724)

# Supplementary methodology

Five other non-invasive scores were investigated in addition to FIB-4 as follows:

- Non-alcoholic fatty liver disease (NAFLD) fibrosis score (NFS)
- Aspartate aminotransferase (AST) to platelet ratio index (APRI)
- Forns index
- Body mass index, AST and alanine aminotransferase (ALT) ratio, and diabetes score (BARD)
- AST to ALT ratio (AAR)

# Supplementary results

## Supplementary analyses (FIB-4 at baseline)

The supplementary analyses of FIB-4 at baseline investigating the age-dependent cut-offs suggested by McPherson et al,^1^ potential reverse causality (by excluding individuals with events occurring in the first 6 months or the first 12 months of follow-up, respectively), and shorter follow-up time of 2·5 years or 5 years are shown in supplementary tables 5–7. Analyses showed higher HRs for both high and indeterminate versus low FIB-4 group for liver events, and higher HRs for high FIB-4 versus low FIB-4 for CV events and all-cause mortality.

Analyses of baseline FIB-4 in individuals with obesity, individuals with T2D, and individuals with both obesity and T2D showed similar results to analyses of individuals with obesity and/or T2D (data not shown).

Analyses of other non-invasive scores (NFS, APRI, Forns index; BARD, and AAR) measured at baseline showed similar results to FIB-4 analyses (see supplementary tables 8 and 9, supplementary figure 2).

## Supplementary analyses (change in FIB-4)

In the supplementary analyses conducted to assess the impact of length of time between repeat FIB-4 measurements, associations between 6-month or 36-month changes in FIB-4 with liver events, CV events, and all-cause mortality, results were similar to results of 12-month changes in FIB-4 (data not shown).

*Supplementary Table 1:* Codes used to identify criteria for exclusion in CPRD, HES ADMIN, and HES OP

| **Diseases** | **CPRD** | **HES ADMIN and HES OP** |
| --- | --- | --- |
|  | **Ingredients** | **ICD10 codes** |
| Genetic liver diseases |  | E83·0, E83·0B, E83·1, E88·0A, E88·0B |
| Budd-Chiari Syndrome |  | I82·0, K76·5 |
| Viral and autoimmune hepatitis  Acute hepatitis A  Acute hepatitis B  Other acute viral hepatitis  Chronic viral hepatitis  Unspecified viral hepatitis  Autoimmune hepatitis |  | B15  B16  B17  B18  B19  K75·4 |
| Biliary liver diseases  Primary biliary cirrhosis  Secondary biliary cirrhosis  Biliary cirrhosis, unspecified  Primary sclerosing cholangitis |  | K74·3  K74·4  K74·5  K83·0 |
| Drug-induced liver disease |  | K71·3–K71·7 |
| Drugs known to induce liver disease | Methotrexate, amiodarone, tamoxifen, fluorouracil, irinotecan, carboplatin, cisplatin, oxaliplatin |  |
| Alcohol-related disorders |  | E24·4, F10, G31·2, G40·05, G62·1, G72·1, I42·6, K29·2, K70, K85·2, K86·0, O35·4 |
| Human immunodeficiency virus disease |  | B20-B24, Z21, R75, F02·4, O98·7 |

Diseases were defined as ICD10 codes from HES OP or HES ADMIN. Drugs known to induce liver diseases were identified by the presence of a prescription record in CPRD. Diseases were defined as present by the presence of a code in HES and assumed to be absent by the absence of a code.

ADMIN=Admitted Patient Care Data. CPRD=Clinical Practice Research Datalink. CV=cardiovascular. HES=Hospital Episodes Statistics. ICD=10^th^ revision of the International Statistical Classification of Diseases and Related Health Problems. ONS=Office for National Statistics. OP=outpatient data.

*Supplementary Table 2:* Codes used to identify liver events, CV events, and all-cause mortality in ONS, HES ADMIN, and HES OP

| **Outcome/definition** | **ONS** | **HES ADMIN and HES OP** | |
| --- | --- | --- | --- |
|  | **ICD10** | **ICD10** | **OPCS4** |
| **Liver event** using the first record of any of the below events captured in HES or ONS | | | |
| Liver death | K70–K77, C22·0 |  |  |
| Hospital contact for hepatocellular carcinoma |  | C22·0 |  |
| Hospital contact for liver transplant |  | Z94·4 | J01 |
| Hospital contact for chronic liver failure |  | K72·1 or K72·9 |  |
| Hospital contact for liver cirrhosis |  | K74·6 |  |
| Hospital contact for portal hypertension |  | K76·6 |  |
| Hospital contact for decompensated liver events: |  |  |  |
| - Ascites |  | R18 | T46·1, T46·2, T46·8, T46·9 |
| - Transjugular intrahepatic portal shunt |  |  | J06·1, J06·2, J06·8, J06·9, J11·4, J11·8, J11·9, L77 |
| - Hepatorenal syndrome |  | K76·7 |  |
| - Hepatic encephalopathy |  | K72·0, K72·1, K72·9, G94·3 |  |
| - Gastro-oesophageal varices with/without bleeding |  | I85, I86·4, I98·2, I98·3 | G10·4, G10·5, G10·8, G10·9, G14·4, G17·4, G43·7 |
| **CV event** using the first record of any of the below events captured in HES or ONS | | | |
| CV death | I00–I99 |  |  |
| Hospital contact for stroke |  | I60–I64 except I60·80 |  |
| Hospital contact for acute myocardial infarction |  | I21 |  |
| Hospital contact for unstable angina |  | I20·0 |  |
| Hospital contact for heart failure |  | I50 |  |
| Hospital contact for coronary revascularization* |  |  | K40–46, K49–50 |
| **Death** using a record in ONS Death Registration Data | | | |
| Death by any cause (ONS) | As recorded |  |  |

Events in each endpoint are defined as ICD10 or OPCS4 codes from HES OP, HES ADMIN, or ONS Death Registration Data. Events were defined as present by the presence of a code in HES or ONS and assumed to be absent by the absence of a code.

ADMIN=Admitted Patient Care Data. CV=cardiovascular. HES=Hospital Episodes Statistics. ICD=10^th^ revision of the International Statistical Classification of Diseases and Related Health Problems. ONS=Office for National Statistics. OP=outpatient data.

*Coronary revascularisation = coronary artery bypass grafting and percutaneous coronary intervention.

*Supplementary Table 3:* Baseline demographics and clinical characteristics in the baseline and 12-month change analyses

| **Baseline parameter** | **Overall FIB-4 population for baseline analyses** | **Overall FIB-4 population for  12 month change analyses** |
| --- | --- | --- |
| **N*** | 44 481 | 20 548^†^ |
| **Patient characteristics**  Female, %  Age, years  BMI, kg/m^2^  T2D, %  T2D duration, years  Framingham risk score^‡^  Smoking status^‡^  Current smoker  Ex-smoker  Never smoker | 54  58·8 (29·5, 83·7)  32·1 (23·8, 43·3)  44  0 (0·0, 12·1)  20·2 (5·5, 54·3)  16  26  35 | 54  62·1 (33·2, 84·6)  31·8 (23·3, 43·0)  52  0 (0·0, 13·0)  21·4 (6·1, 55·3)  15  27  35 |
| **Liver parameters**  AST, U/L  ALT, U/L | 24 (15, 53)  26 (12, 78) | 23 (15, 52)  25 (11, 75) |
| **Metabolic parameters**  HbA_1c_^‡^, %  Creatinine, µmol/L  HDL^‡^, mmol/L  LDL^‡^, mmol/L  Triglycerides^‡^, mmol/L | 6·9 (5·5, 10·8)  83 (57, 127)  1·2 (0·8, 2·0)  2·9 (1·4, 4·8)  1·6 (0·7, 3·9) | 7·0 (5·5, 10·7)  84 (58, 133)  1·2 (0·8, 2·0)  2·7 (1·4, 4·7)  1·6 (0·7, 3·9) |
| **Hospitalisation for comorbidities, %**  Hypertension  Dyslipidaemia  CKD | 53  27  13 | 60  31  16 |
| **Prescribed medication, %**  Anti-hypertensive  Metformin  Statin | 31  19  34 | 36  23  41 |

Values are median (5^th^ percentile, 95^th^ percentile) unless otherwise stated.

ALT=alanine aminotransferase. AST=aspartate aminotransferase. BMI=body mass index. CKD=chronic kidney disease. FIB-4=Fibrosis-4 Index. HbA_1c_=glycated haemoglobin. HDL=high-density lipoprotein. LDL=low-density lipoprotein. T2D=type 2 diabetes.

*Individuals were followed from baseline, ie, their first FIB-4 measurement after 1 January 2001 (baseline analyses) or from their second FIB-4 measurement after 1 January 2001 (12-month change analyses).

^†^Two individuals experienced events on the day of the second FIB-4 measurement and were excluded from analyses of liver events, cardiovascular events and all-cause mortality.

^‡^Missing for ≥20% of the population.

*Supplementary Table 4:* Baseline demographics and clinical characteristics (GP observed data*)

| **Baseline parameter** | **FIB-4 low**  **(<1·30)** | **FIB-4 indeterminate (1·30‒2·67)** | **FIB-4 high**  **(>2·67)** | **Overall FIB-4 population** |
| --- | --- | --- | --- | --- |
| **N** | 90 841 | 39 454 | 7113 | 137 408 |
| **Patient characteristics**  Female, %  Age, years  BMI, kg/m^2^  T2D, %  T2D duration, years  Framingham risk score^†^  Smoking status^†^  Current smoker  Ex-smoker  Never smoker | 57  51·8 (26·1, 75·8)  32·8 (25·7, 44·6)  35  0 (0·0, 10·0)  18·5 (5·0, 52·8)  19  22  36 | 46  70·0 (50·0, 86·6)  31·5 (23·1, 41·5)  54  0 (0·0, 15·1)  24·2 (9·0, 57·9)  11  31  36 | 41  72·4 (48·5, 89·7)  31·0 (21·9, 41·2)  63  0·4 (0·0, 16·5)  22·8 (8·0, 58·4)  12  30  34 | 53  58·4 (28·9, 82·9)  32·4 (24·4, 43·7)  42  0 (0·0, 12·2)  20·4 (5·7, 54·7)  16  25  36 |
| **Liver parameters**  AST, U/L  ALT, U/L | 22 (13, 43)  26 (12, 74) | 25 (16, 68)  24 (11, 89) | 44 (19, 251)  33 (10, 266) | 23 (14, 59)  26 (11, 83) |
| **Metabolic parameters**  HbA_1c_^†^, %  Creatinine, µmol/L  HDL^†^, mmol/L  LDL^†^, mmol/L  Triglycerides^†^, mmol/L | 7·1 (5·5, 11·1)  77 (55, 114)  1·2 (0·8, 1·9)  3·0 (1·5, 4·9)  1·7 (0·8, 4·3) | 6·8 (5·5, 10·0)  87 (59, 143)  1·2 (0·8, 2·0)  2·5 (1·2, 4·5)  1·6 (0·7, 3·9) | 6·6 (5·2, 9·9)  86 (55, 167)  1·2 (0·7, 2·2)  2·3 (1·0, 4·3)  1·4 (0·7, 3·8) | 6·9 (5·4, 10·7)  80 (56, 126)  1·2 (0·8, 1·9)  2·8 (1·3, 4·7)  1·6 (0·8, 4·1) |
| **Prescribed medication, %**  Anti-hypertensive  Metformin  Statin | 25  17  29 | 47  22  53 | 52  24  50 | 33  19  37 |

Values are median (5^th^ percentile, 95^th^ percentile) unless otherwise stated.

ALT=alanine aminotransferase. AST=aspartate aminotransferase. BMI=body mass index. FIB-4=Fibrosis-4 Index. GP=general practitioner. HbA_1c_=glycated haemoglobin. HDL=high-density lipoprotein. LDL=low-density lipoprotein. T2D=type 2 diabetes.

*Observation period derived from the GP data.

^†^Missing for ≥20% of the population.

*Supplementary Table 5:* Hazard ratios of liver events, CV events, and all-cause mortality by baseline FIB-4 using age-dependent FIB-4 cut-offs

| **FIB-4 category** | **Patients  (n)** | **Events  (n)** | **Crude HR  (95% CI)** | **Adjusted  (age and sex)  HR (95% CI)** |
| --- | --- | --- | --- | --- |
| **Liver events**  Overall  FIB-4 low  FIB-4 indeterminate  FIB-4 high | 40 188  31 954*  6373  1861 | 952  457  233  262 | 1·00  2·68 (2·29–3·14)  14·25 (12·23–16·59) | 1·00  2·46 (2·09–2·89)  12·40 (10·51–14·64) |
| **CV events**  Overall  FIB-4 low  FIB-4 indeterminate  FIB-4 high | 36 445  29 267*  5621  1557 | 5961  4386  1088  487 | 1·00  1·36 (1·27–1·45)  2·95 (2·68–3·24) | 1·00  1·01 (0·95–1·08)  1·33 (1·21–1·47) |
| **All-cause mortality**  Overall  FIB-4 low  FIB-4 indeterminate  FIB-4 high | 40 347  32 011*  6409  1927 | 8941  6220  1631  1090 | 1·00  1·36 (1·29–1·44)  4·10 (3·84–4·37) | 1·00  0·98 (0·93–1·04)  1·58 (1·48–1·69) |

HRs and 95% CI were estimated using Cox proportional hazard models with time since first FIB-4 measurement as the underlying timescale. Crude results and results adjusted for sex and age at baseline are presented. FIB-4 score at baseline was categorised as low (<1·30), indeterminate (1·30‒2·67), or high (>2·67) based on risk of advanced fibrosis.

CI=confidence interval. CV=cardiovascular. FIB-4=Fibrosis-4 Index. HR=hazard ratio.

*One individual in the FIB-4 low group had no information on sex and was therefore excluded from age- and sex-adjusted analyses.

*Supplementary Table 6:* Hazard ratios of liver events, CV events, and all-cause mortality by baseline FIB-4, excluding patients with events occurring in the first 6 months or first 12 months

|  | **Excluding patients with events occurring in the first 6 months** | | | | **Excluding patients with events occurring in the first 12 months** | | | |
| --- | --- | --- | --- | --- | --- | --- | --- | --- |
|  | **Patients  (n)** | **Events  (n)** | **Crude HR  (95% CI)** | **Adjusted  (age and sex)  HR (95% CI)** | **Patients  (n)** | **Events  (n)** | **Crude HR  (95% CI)** | **Adjusted  (age and sex)  HR (95% CI)** |
| **Liver events**  Overall  FIB-4 low  FIB-4 indeterminate  FIB-4 high | 43 513  29 014*  12 812  1687 | 864  311  349  204 | 1·00  2·91 (2·49–3·39)  16·37 (13·71–19·54) | 1·00  2·51 (2·10–2·99)  14·37 (11·72–17·62) | 42 890  28 774  12 519  1597 | 807  297  332  178 | 1·00  2·92 (2·49–3·41)  15·34 (12·73–18·48) | 1·00  2·51 (2·09–3·01)  13·41 (10·83–16·61) |
| **CV events**  Overall  FIB-4 low  FIB-4 indeterminate  FIB-4 high | 39 715  27 294*  10 995  1426 | 5500  2490  2591  419 | 1·00  3·00 (2·84–3·17)  4·56 (4·11–5·06) | 1·00  1·02 (0·96–1·09)  1·30 (1·17–1·46) | 39 049  27 027  10 678  1344 | 5122  2353  2396  373 | 1·00  2·96 (2·80–3·14)  4·41 (3·95–4·92) | 1·00  1·01 (0·95–1·08)  1·27 (1·14–1·43) |
| **All-cause mortality**  Overall  FIB-4 low  FIB-4 indeterminate  FIB-4 high | 43 734  29 079*  12 875  1780 | 8228  3074  4216  938 | 1·00  3·52 (3·36–3·69)  7·03 (6·53–7·56) | 1·00  1·00 (0·95–1·05)  1·52 (1·41–1·65) | 43 129  28 842  12 591  1696 | 7666  2868  3940  858 | 1·00  3·56 (3·39–3·73)  7·07 (6·55–7·63) | 1·00  1·01 (0·96–1·06)  1·54 (1·42–1·67) |

HRs and 95% CI were estimated using Cox proportional hazard models with time since first FIB-4 measurement as the underlying timescale. Crude results and results adjusted for sex and age at baseline are presented. FIB-4 score at baseline was categorised as low (<1·30), indeterminate (1·30‒2·67), or high (>2·67) based on risk of advanced fibrosis.

CI=confidence interval. CV=cardiovascular. FIB-4=Fibrosis-4 Index. HR=hazard ratio.

*One individual in the FIB-4 low group had no information on sex and was therefore excluded from age- and sex-adjusted analyses.

*Supplementary Table 7:* Hazard ratios of liver events, CV events, and all-cause mortality by baseline FIB-4 with 2·5 years or 5 years follow-up

|  | **2·5 years follow-up** | | | | **5 years follow-up** | | | |
| --- | --- | --- | --- | --- | --- | --- | --- | --- |
|  | **Patients  (n)** | **Events  (n)** | **Crude HR  (95% CI)** | **Adjusted  (age and sex)  HR (95% CI)** | **Patients  (n)** | **Events  (n)** | **Crude HR  (95% CI)** | **Adjusted  (age and sex)  HR (95% CI)** |
| **Liver events**  Overall  FIB-4 low  FIB-4 indeterminate  FIB-4 high | 44 311  29 307*  13 138  1866 | 346  92  106  148 | 1·00  2·65 (2·01–3·51)  29·07 (22·41–37·72) | 1·00  2·54 (1·85–3·47)  29·38 (21·63–39·91) | 44 311  29 307  13 138  1866 | 572  163  209  200 | 1·00  3·05 (2·49–3·75)  24·44 (19·87–30·06) | 1·00  2·80 (2·21–3·53)  23·46 (18·40–29·90) |
| **CV events**  Overall  FIB-4 low  FIB-4 indeterminate  FIB-4 high | 40 565  27 660*  11 342  1563 | 1929  766  944  219 | 1·00  3·13 (2·85–3·44)  5·80 (4·99–6·74) | 1·00  1·02 (0·91–1·13)  1·50 (1·28–1·77) | 40 565  27 660  11 342  1563 | 3467  1444  1682  341 | 1·00  3·08 (2·87–3·30)  5·28 (4·69–5·94) | 1·00  1·01 (0·93–1·09)  1·41 (1·24–1·60) |
| **All-cause mortality**  Overall  FIB-4 low  FIB-4 indeterminate  FIB-4 high | 44 477  29 355*  13 189  1933 | 2838  1035  1341  462 | 1·00  2·98 (2·75–3·23)  7·67 (6·87–8·55) | 1·00  0·82 (0·75–0·89)  1·53 (1·36–1·73) | 44 477  29 355  13 189  1933 | 5067  1819  2510  738 | 1·00  3·28 (3·09–3·48)  7·63 (7·01–8·32) | 1·00  0·91 (0·85–0·97)  1·56 (1·42–1·71) |

HRs and 95% CI were estimated using Cox proportional hazard models with time since first FIB-4 measurement as the underlying timescale. Crude results and results adjusted for sex and age at baseline are presented. FIB-4 score at baseline was categorised as low (<1·30), indeterminate (1·30‒2·67), or high (>2·67) based on risk of advanced fibrosis.

CI=confidence interval. CV=cardiovascular. FIB-4=Fibrosis-4 Index. HR=hazard ratio.

*One individual in the FIB-4 low group had no information on sex and was therefore excluded from age- and sex-adjusted analyses.

*Supplementary Table 8:* Hazard ratios of liver events, CV events, and all-cause mortality by baseline non-invasive score

|  | **Patients  (n)*** | **Events  (n)** | **Crude HR  (95% CI)** | | **Adjusted  (age and sex)  HR (95% CI)** |
| --- | --- | --- | --- | --- | --- |
| ***APRI*** | | | | | |
| **Liver events**  APRI low  APRI indeterminate  APRI high  **CV events**  APRI low  APRI indeterminate  APRI high  **All-cause mortality**  APRI low  APRI indeterminate  APRI high | 120 719  8285  998  111 141  7636  940  120 969  8365  1029 | 1571  691  228  16 278  1161  146  23 165  1950  413 | 1·00  6·81 (6·23–7·45)  23·67 (20·60–27·20)  1·00  1·08 (1·02–1·15)  1·30 (1·11–1·54)  1·00  1·27 (1·21–1·33)  2·59 (2·35–2·85) | | 1·00  6·66 (6·08–7·30)  23·39 (20·34–26·89)  1·00  1·10 (1·04–1·17)  1·34 (1·13–1·57)  1·00  1·41 (1·35–1·48)  2·69 (2·44–2·97) |
| ***Forns index*** | | | | | |
| **Liver events**  Forns low  Forns indeterminate  Forns high  **CV events**  Forns low  Forns indeterminate  Forns high  **All-cause mortality**  Forns low  Forns indeterminate  Forns high | 60 242  78 265  13 571  58 652  69 325  10 455  60 321  78 469  13 773 | 515  1541  1331  4405  13 437  3430  4230  18 681  6796 | 1·00  2·53 (2·29–2·79)  16·16 (14·60–17·90)  1·00  2·89 (2·80–3·00)  6·23 (5·96–6·51)  1·00  3·73 (3·60–3·85)  9·77 (9·41–10·16) | | 1·00  2·78 (2·48–3·12)  20·25 (17·78–23·05)  1·00  1·15 (1·11–1·20)  1·53 (1·45–1·61)  1·00  1·21 (1·16–1·25)  1·80 (1·72–1·88) |
| ***BARD*** | | | | | |
| **Liver events**  BARD low  BARD high  **CV events**  BARD low  BARD high  **All-cause mortality**  BARD low  BARD high | 14 681  37 381*  13 983  34 133*  14 703  37 528* | 159  922  1095  5729  1184  8112 | 1·00  2·41 (2·03–2·85)  1·00  2·29 (2·14–2·44)  1·00  2·84 (2·67–3·02) | | 1·00  2·09 (1·76–2·49)  1·00  1·33 (1·24–1·42)  1·00  1·31 (1·23–1·40) |
| ***NFS*** | | | | | |
| **Liver events**  NFS low  NFS indeterminate  NFS high  **CV events**  NFS low  NFS indeterminate  NFS high  **All-cause mortality**  NFS low  NFS indeterminate  NFS high | 20 125*  18 355  3583  19 321*  16 430  2913  20 160*  18 410  3646 | 223  429  261  1346  3208  1010  1599  4470  1938 | | 1·00  2·29 (1·95–2·69)  9·13 (7·63–10·92)  1·00  3·12 (2·93–3·32)  7·15 (6·59–7·76)  1·00  3·33 (3·14–3·52)  9·39 (8·79–10·04) | 1·00  1·80 (1·50–2·15)  6·98 (5·67–8·60)  1·00  1·29 (1·21–1·39)  1·86 (1·69–2·03)  1·00  1·11 (1·05–1·18)  1·76 (1·64–1·90) |
| ***AAR*** | | | | | |
| **Liver events**  AAR low  AAR high  **CV events**  AAR low  AAR high  **All-cause mortality**  AAR low  AAR high | 32 580  21 483*  30 512  19 252*  32 658  21 582* | 540  609  3436  3811  4018  6269 | | 1·00  1·88 (1·68–2·11)  1·00  1·94 (1·85–2·03)  1·00  2·60 (2·50–2·71) | 1·00  1·75 (1·54–1·98)  1·00  1·22 (1·16–1·28)  1·00  1·35 (1·29–1·41) |

HRs and 95% CI were estimated using Cox proportional hazard models with time since first FIB-4 measurement as the underlying timescale.. Crude results and results adjusted for sex and age at baseline are presented. Non-invasive scores at baseline were categorised based on risk of advanced fibrosis as follows: APRI: low (<0·5), indeterminate (0·5–1·5), or high (>1·5); Forns: low (≥4·2), indeterminate (>4·2–6·9), or high (≥6·9); BARD: low (0–1) or high (2–4); NFS: low (<–1·455), indeterminate (–1·455–0·676), or high (>0·676); AAR: low (<1) or high (≥1).

AAR=aspartate aminotransferase to alanine aminotransferase ratio. APRI=aspartate aminotransferase to platelet ratio index. BARD=body mass index, aspartate aminotransferase and alanine aminotransferase ratio and diabetes score. CI=confidence interval. CV=cardiovascular. HR=hazard ratio. NFS=non-alcoholic fatty liver disease fibrosis score.

*One individual in the BARD high, NFS low, and AAR high groups had no information on sex and was therefore excluded from age- and sex-adjusted analyses.

*Supplementary Table 9:* Hazard ratios of liver events, CV events, and all-cause mortality by baseline non-invasive scores in the subpopulations with a Framingham risk score available

|  | | **Patients  (n)** | **Events  (n)** | **Crude HR  (95% CI)** | **Adjusted  (age, sex)  HR (95% CI)** | **Adjusted  (age, sex, FramH) HR (95% CI)** |
| --- | --- | --- | --- | --- | --- | --- |
| ***APRI*** | | | | | | |
| **Liver events**  APRI low  APRI indeterminate  APRI high  **CV events**  APRI low  APRI indeterminate  APRI high  **All-cause mortality**  APRI low  APRI indeterminate  APRI high | | 47 019  3608  315  43 467  3330  305  47 101  3628  328 | 593  309  81  5129  417  43  5386  595  102 | 1·00  7·25 (6·32–8·32)  26·84 (21·28–33·86)  1·00  1·11 (1·00–1·22)  1·45 (1·07–1·96)  1·00  1·50 (1·38–1·63)  3·30 (2·71–4·02) | 1·00  7·19 (6·25–8·27)  25·81 (20·45–32·58)  1·00  1·03 (0·93–1·14)  1·34 (0·99–1·81)  1·00  1·46 (1·34–1·59)  3·09 (2·54–3·76) | 1·00  7·17 (6·23–8·25)  25·72 (20·37–32·46)  1·00  1·02 (0·92–1·13)  1·29 (0·95–1·74)  1·00  1·45 (1·33–1·58)  3·06 (2·52–3·72) |
| ***Forns index*** | | | | | | |
| **Liver events**  Forns low  Forns indeterminate  Forns high  **CV events**  Forns low  Forns indeterminate  Forns high  **All-cause mortality**  Forns low  Forns indeterminate  Forns high | | 35 373  42 328  4982  34 455  37 973  4025  35 421  42 435  5071 | 298  848  644  2450  5559  990  2018  6021  1598 | 1·00  2·49 (2·18–2·84)  18·86 (16·44–21·64)  1·00  2·21 (2·10–2·31)  4·33 (4·02–4·66)  1·00  2·61 (2·48–2·75)  6·69 (6·27–7·15) | 1·00  2·85 (2·46– 3·31)  24·17 (20·42–28·62)  1·00  1·17 (1·11–1·23)  1·70 (1·56–1·84)  1·00  1·24 (1·17–1·31)  2·27 (2·10–2·45) | 1·00  2·89 (2·49–3·35)  25·54 (21·59–30·21)  1·00  1·26 (1·20–1·33)  2·02 (1·85–2·19)  1·00  1·31 (1·24–1·39)  2·58 (2·39–2·78) |
| ***BARD*** | | | | | | |
| **Liver events**  BARD low  BARD high  **CV events**  BARD low  BARD high  **All-cause mortality**  BARD low  BARD high | | 6608  15 969  6230  14 651  6619  16 031 | 67  432  506  1988  416  2231 | 1·00  2·71 (2·09–3·50)  1·00  1·71 (1·55–1·89)  1·00  2·25 (2·02–2·50) | 1·00  2·58 (1·98–3·35)  1·00  1·39 (1·26–1·54)  1·00  1·60 (1·44–1·78) | 1·00  2·60 (1·99–3·38)  1·00  1·43 (1·30–1·59)  1·00  1·63 (1·46–1·81) |
| ***NFS*** | | | | | | |
| **Liver events**  NFS low  NFS indeterminate  NFS high  **CV events**  NFS low  NFS indeterminate  NFS high  **All-cause mortality**  NFS low  NFS indeterminate  NFS high | 8316  8337  1133  7859  7495  976  8332  8364  1163 | | 92  196  121  624  1125  255  614  1264  385 | 1·00  2·19 (1·71–2·81)  11·46 (8·74–15·03)  1·00  1·99 (1·80–2·19)  4·03 (3·48–4·66)  1·00  2·12 (1·92–2·33)  5·33 (4·69–6·05) | 1·00  2·00 (1·54–2·60)  10·30 (7·68–13·83)  1·00  1·26 (1·14–1·40)  2·19 (1·88–2·55)  1·00  1·16 (1·05–1·29)  2·39 (2·09–2·73 | 1·00  2·00 (1·54–2·60)  10·31 (7·68–13·83)  1·00  1·28 (1·16–1·42)  2·24 (1·92–2·61)  1·00  1·17 (1·06–1·30)  2·42 (2·12–2·77) |
| ***AAR*** | | | | | | |
| **Liver events**  AAR low  AAR high  **CV events**  AAR low  AAR high  **All-cause mortality**  AAR low  AAR high | 14 554  7508  13 564  6820  14 583  7547 | | 248  233  1378  1017  1280  1281 | 1·00  1·88 (1·57–2·24)  1·00  1·52 (1·40–1·65)  1·00  2·00 (1·85–2·16) | 1·00  1·87 (1·55–2·25)  1·00  1·36 (1·25–1·48)  1·00  1·60 (1·48–1·73) | 1·00  1·86 (1·54–2·24)  1·00  1·34 (1·23–1·46)  1·00  1·58 (1·46–1·72) |

HRs and 95% CI were estimated using Cox proportional hazard models with time since first FIB-4 measurement as the underlying timescale. Crude results, results adjusted for sex and age at baseline, and results adjusted for sex, age, and Framingham CV risk score are presented. Adjustment for Framingham CV risk score was at the time of non-invasive score baseline measurement. Non-invasive scores at baseline were categorised based on risk of advanced fibrosis as follows: APRI: low (<0·5), indeterminate (0·5–1·5), or high (>1·5); Forns: low (≥4·2), indeterminate (>4·2–6·9), or high (≥6·9); BARD: low (0–1) or high (2–4); NFS: low (<–1·455), indeterminate (–1·455–0·676), or high (>0·676); AAR: low (<1) or high (≥1).

AAR=aspartate aminotransferase to alanine aminotransferase ratio. APRI=aspartate aminotransferase to platelet ratio index. BARD=body mass index, aspartate aminotransferase and alanine aminotransferase ratio and diabetes score. CI=confidence interval. CV=cardiovascular. FramH=Framingham. HR=hazard ratio. NFS=non-alcoholic fatty liver disease fibrosis score.

*Supplementary Table 10:* Hazard ratios of liver events for 12-month, 0·5 unit increase in FIB-4.

| **FIB-4 category** | **Patients  (n)** | **Events  (n)** | **Crude HR  (95% CI)** | **Adjusted  (age and sex)  HR (95% CI)** |
| --- | --- | --- | --- | --- |
| **Liver events**  Overall  FIB-4 low  FIB-4 indeterminate  FIB-4 high | 20 443  12 653  6893  897 | 466  163  202  101 | 1·57 (1·44–1·73)  3·65 (2·93–4·55)  19·92 (15·12–26·25) | 1·58 (1·43–1·74)  3·50 (2·72–4·50)  19·87 (14·53–27·17) |

Reference is patients with low baseline FIB-4 and no change in FIB-4 (HR=1). Time since FIB-4 measurement as the underlying timescale and age included in strata (baseline hazard). The model included: change in FIB-4 (continuous), baseline FIB-4 (categorical), sex (categorical), and the interaction change in FIB-4 (continuous)*baseline FIB-4 (categorical). FIB-4 risk categories: low <1·30; indeterminate 1·30–2·67; high >2·67.

CI=confidence interval. FIB-4=Fibrosis-4 Index. HR=hazard ratio.

*Supplementary Figure 1:* Cumulative incidence of (A) CV events and (B) all-cause mortality according to a 12-month increase or decrease in FIB-4 by baseline FIB-4 category


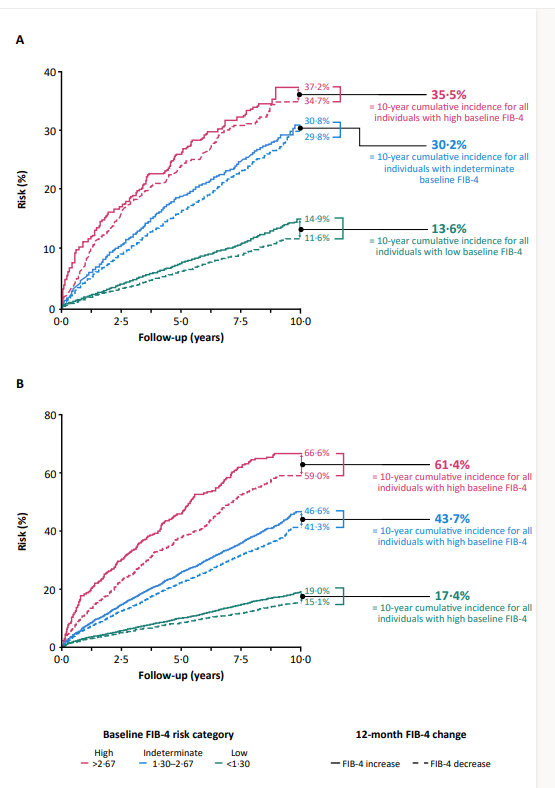


Event risks plotted as Aalen-Johansen cumulative incidence functions, with all-cause mortality included as a competing risk for CV events. FIB-4 risk categories: low <1·30; indeterminate 1·30–2·67; high >2·67.

CV=cardiovascular. FIB-4=Fibrosis-4 Index.

***Supplementary Figure 2:* Cumulative incidence according to baseline non-invasive score for A) APRI, B) Forns, C) BARD, D) NFS, E) AAR**


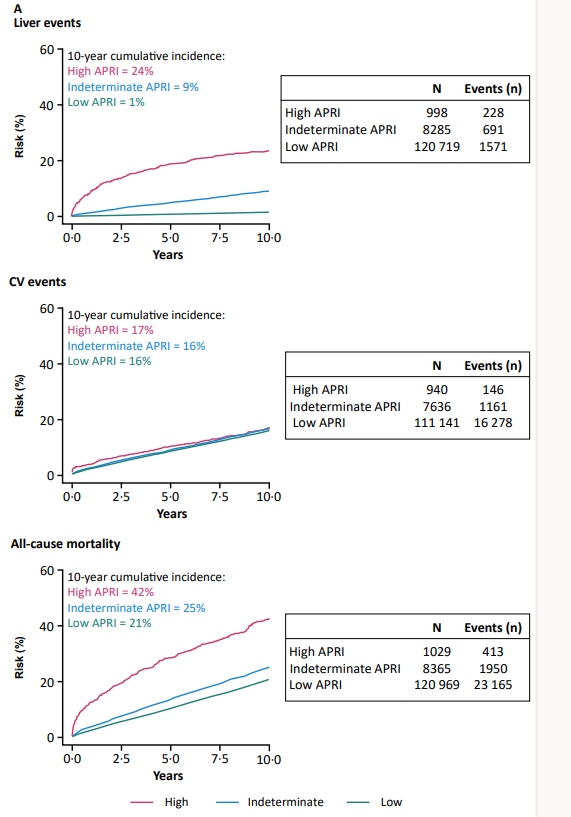


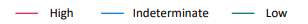

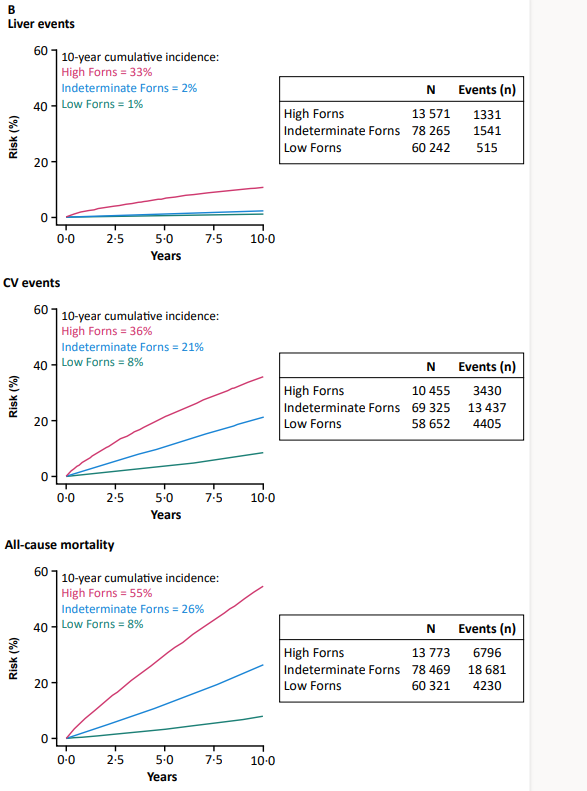


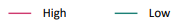

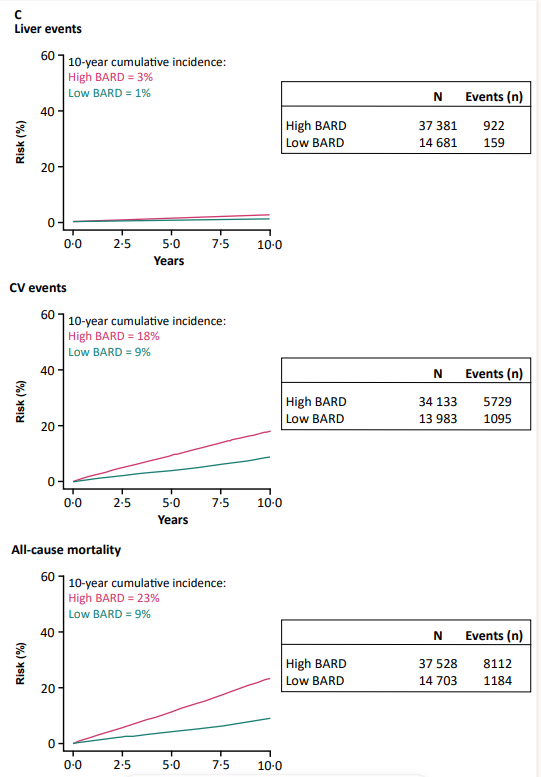


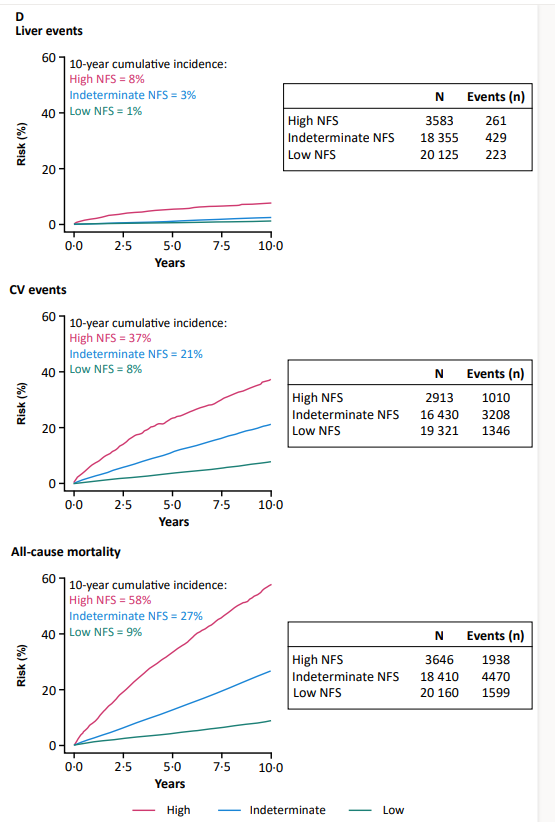


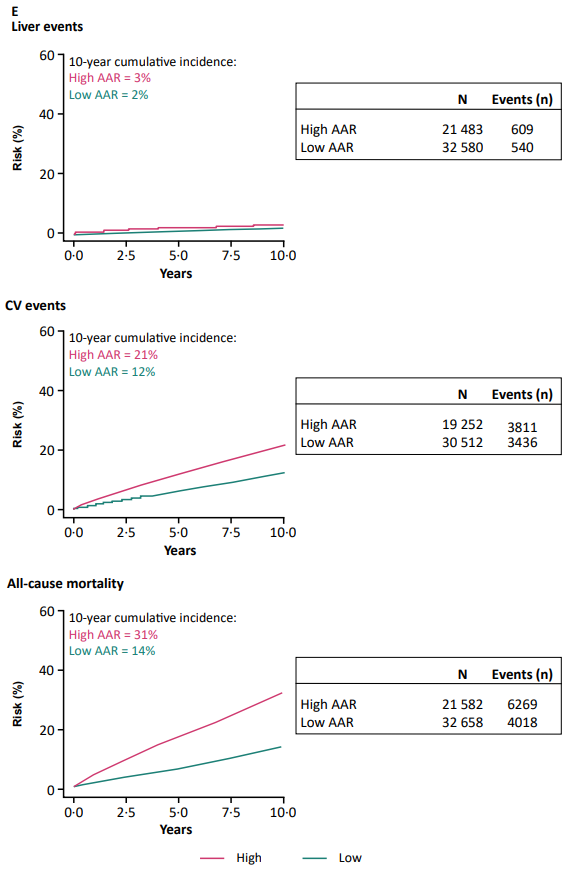


Event risks plotted as Aalen-Johansen cumulative incidence functions, with all-cause mortality included as a competing risk factor in plots of liver and CV events. APRI risk categories: low (<0·5), indeterminate (0·5–1·5), or high (>1·5); Forns risk categories: low (≥4·2), indeterminate (>4·2–6·9), or high (≥6·9); BARD risk categories: low (0–1) or high (2–4); NFS risk categories: low (<–1·455), indeterminate (–1·455–0·676), or high (>0·676); AAR risk categories: low (<1) or high (≥1).

AAR=aspartate aminotransferase to alanine aminotransferase ratio. APRI=aspartate aminotransferase to platelet ratio index. BARD=body mass index, aspartate aminotransferase and alanine aminotransferase ratio and diabetes score. CV=cardiovascular. NFS=non-alcoholic fatty liver disease fibrosis score.

# Supplementary reference

1. McPherson S, Hardy T, Dufour JF, et al. Age as a confounding factor for the accurate non-invasive diagnosis of advanced NAFLD fibrosis. *Am J Gastroenterol* 2017;**112:**740–51.
