# Supplementary material for: Prognostic utility of Fibrosis-4 Index for risk of subsequent liver and cardiovascular events, and all-cause mortality in individuals with obesity and/or type 2 diabetes: a longitudinal cohort study
Source: Lancet Reg Health Eur. 2023 Dec 19;36:100780. doi: 10.1016/j.lanepe.2023.100780 (PMC10769893; doi:10.1016/j.lanepe.2023.100780)
Supplement: Graphical abstract [file mmc3.pdf]

Visual abstract

Prognostic utility of Fibrosis-4 Index for risk of subsequent liver and cardiovascular events, and all-cause mortality in individuals with obesity and/or type 2 diabetes: a longitudinal cohort study

Anstee QM, Berentzen TL, Nitze LM, et al.

1 The study

Participants: Adults aged ≥18 years with obesity and/or type 2 diabetes and ≥1 FIB-4 score calculable from UK Clinical Practice Research Datalink (CPRD) GOLD after 1 January 2001.

Primary outcome: Three composite endpoints: (1) time to first liver event, (2) time to first cardiovascular event and (3) time to all-cause mortality.

Individuals were followed until time of first event, 10 years or 1 January 2020.

Key limitation: The possibility of residual confounding and/or confounding from other factors cannot be fully excluded.

2 Findings

FIB-4 was associated with risk of subsequent liver and cardiovascular events, and all-cause mortality

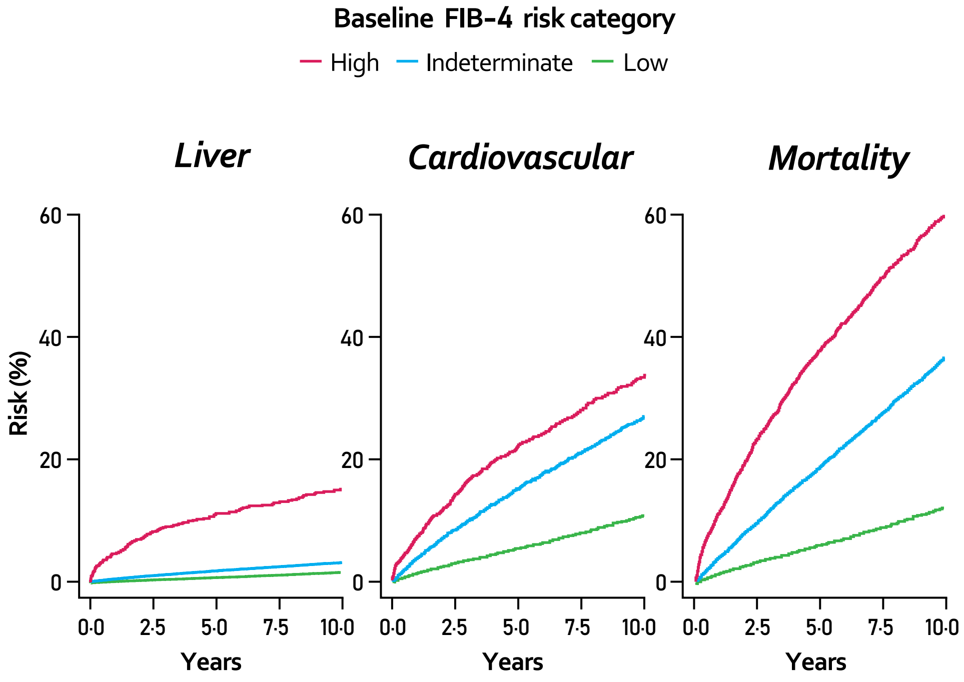

Adjusted HRs for high vs low FIB-4 groups*

Liver events: *16·46 (95% CI 13·65–19·85)*

Cardiovascular events: *1·34 (95% CI 1·21–1·48)*

All-cause mortality: *1·56 (95% CI 1·45–1·68)*

A 12-month increase/decrease in FIB-4 was associated with higher/lower risk of liver and cardiovascular events and all-cause mortality

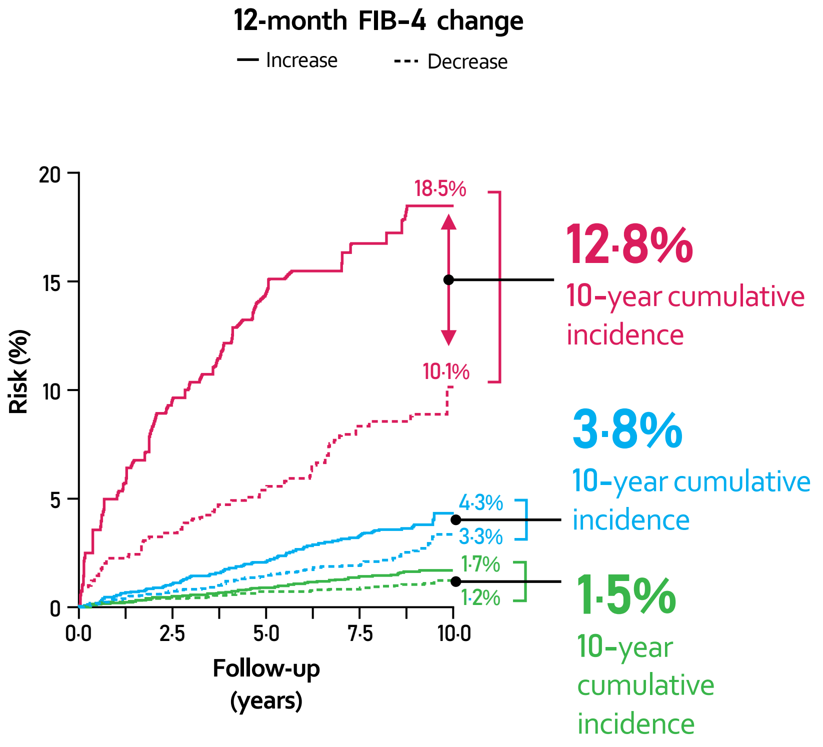

3 Research in context

Clinical guidelines recommend the FIB-4 as a diagnostic biomarker to determine the presence of advanced liver fibrosis in individuals with MASLD or at-risk of MASLD

This real-world study supports the prognostic utility of the FIB-4, with a high risk of incident liver events in groups with FIB-4 scores indicating high and indeterminate risk of advanced fibrosis

FIB-4 has utility in general practice as a prognostic biomarker for risk of subsequent liver and cardiovascular events, and mortality

*HRs and 95% CI were estimated using Cox proportional hazard models with time since first FIB-4 measurement as the underlying timescale. Results adjusted for sex and age at baseline are presented. This study is based in part on data from the CPRD obtained under license from the UK Medicines and Healthcare Products Regulatory Agency. The data are provided by patients and collected by the National Health Service as part of their care and support. ONS provided the ONS data contained within the CPRD data. The interpretation and conclusions contained in this study are those of the authors alone. HES/ONS data (Copyright © 2022) were re-used with the permission of The Health & Social Care Information Centre; all rights reserved. CI, confidence interval; FIB-4, Fibrosis-4 Index; MASLD, metabolic dysfunction-associated steatotic liver disease.
