# Supplementary material for: Prognostic utility of Fibrosis-4 Index for risk of subsequent liver and cardiovascular events, and all-cause mortality in individuals with obesity and/or type 2 diabetes: a longitudinal cohort study
Source: Lancet Reg Health Eur. 2023 Dec 19;36:100780. doi: 10.1016/j.lanepe.2023.100780 (PMC10769893; doi:10.1016/j.lanepe.2023.100780)
Supplement: Cover letter [file mmc2.docx]

Prof Quentin M. Anstee, PhD, FRCP

Translational and Clinical Research Institute,

The Medical School, Newcastle University,

4th Floor, William Leech Building, Framlington Place,

Newcastle-upon-Tyne, NE2 4HH, United Kingdom

Pooja Jha

Editor-in-Chief

The Lancet Regional Health – Europe

[gastrohep@lancet.com](mailto:gastrohep@lancet.com)

Dear Pooja,

**Re: Fibrosis-4 Index is associated with subsequent risk of liver events, cardiovascular events and all-cause mortality in individuals with obesity and/or type 2 diabetes: results from a longitudinal cohort study**

I am writing to submit the aforementioned manuscript for consideration by *The Lancet Regional Health – Europe*. This study investigated the use of FIB-4 as a non-invasive prognostic and monitoring tool for poor clinical outcomes in patients with type 2 diabetes (T2D) and/or obesity.

We conducted a longitudinal cohort study of individuals with T2D and/or obesity who had measurements available for the calculation of FIB-4 during routine primary care. We analysed the correlation between FIB-4 baseline scores and subsequent liver events, cardiovascular events, and all-cause mortality, and further examined whether assessment of change in FIB-4 score over a 12-month interval was associated with these clinical events.

Complementing the existing evidence on the diagnostic utility of FIB-4, our study found that FIB-4 was strongly associated with risk of subsequent liver and CV events, and all-cause mortality, and that changes in FIB-4 further refined the prognostic potential, as shown by the association between 12-month increase/decrease in FIB-4 with higher/lower risk of clinical events, respectively, regardless of patients’ FIB-4 score at baseline.

Our findings have important implications for the clinical management of individuals with, or at risk of, non-alcoholic fatty liver disease (e.g. those with T2D and/or obesity). Our results are in accordance with current guidance recommending use of FIB-4 to aid risk stratification, and suggest that sequential testing of FIB-4 should be incorporated into routine primary care practices for monitoring the long-term risk of adverse outcomes.

We believe that our study makes a valuable contribution to the field, and we would be honoured to have it published in *The Lancet Regional Health – Europe*. Thank you for your consideration.

Sincerely,

Professor Quentin Anstee
